# Design of high-temperature *f*-block molecular nanomagnets through the control of vibration-induced spin relaxation†
†Electronic supplementary information (ESI) available. See DOI: 10.1039/c9sc03133b


**DOI:** 10.1039/c9sc03133b

**Published:** 2019-12-02

**Authors:** Luis Escalera-Moreno, José J. Baldoví, Alejandro Gaita-Ariño, Eugenio Coronado

**Affiliations:** a Instituto de Ciencia Molecular (ICMol) , Universitat de València , c/ Catedrático José Beltrán 2 , Paterna , 46980 , Spain . Email: j.jaime.baldovi@uv.es

## Abstract

One of the main roadblocks that still hamper the practical use of molecular nanomagnets is their cryogenic working temperature. In the pursuit of rational strategies to design new molecular nanomagnets with increasing blocking temperature, *ab initio* methodologies play an important role by guiding synthetic efforts at the lab stage. Nevertheless, when evaluating vibration-induced spin relaxation, these methodologies are still far from being computationally fast enough to provide a useful predictive framework. Herein, we present an inexpensive first-principles method devoted to evaluating vibration-induced spin relaxation in molecular *f*-block single-ion magnets, with the important advantage of requiring only one CASSCF calculation. The method is illustrated using two case studies based on uranium as the magnetic centre. Finally, we propose chemical modifications in the ligand environment with the aim of suppressing spin relaxation.

## Abstract 2

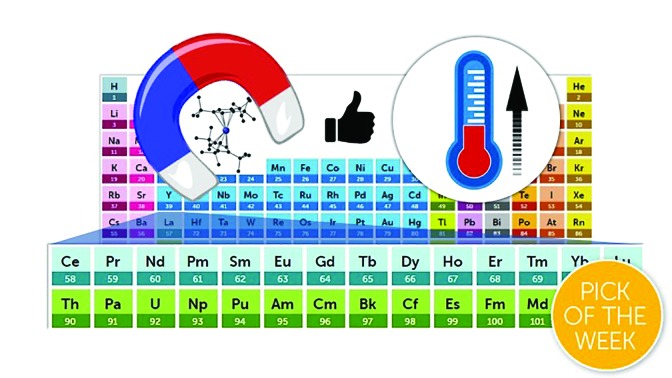
An efficient general first-principles methodology to simulate vibration-induced spin relaxation in *f*-block molecular nanomagnets that drastically reduces the computation time.

## Introduction

Molecular nanomagnets have been attracting enormous attention for almost three decades due to their unique properties. These magnetic molecules, also known as single-molecule magnets (SMMs),^1^ exhibit a bistable ground state that results in a memory effect characterized by a hysteresis loop and have the potential to be harnessed for classical information storage. The second generation of SMMs,^2^ commonly called single-ion magnets (SIMs), is based on coordination complexes with a central magnetic ion as the source of magnetic anisotropy and represents the ultimate limit of miniaturization. Two key parameters are used to characterize the performance of SMMs and SIMs, namely, magnetic relaxation time and blocking temperature. The former is the timescale in which molecular nanomagnets preserve classical information at a given temperature, and the latter gives the maximum temperature that allows observing magnetic hysteresis.

The field of molecular nanomagnetism is at a crucial point. Over the last few years, we have witnessed the discovery of new SIMs that have allowed an outstanding increase in the blocking temperature, first from 30 K to 60 K (2017)^3^,^4^ and then up to 80 K (2018).^5^ This trend is drawing unprecedented interest from many researchers around the world and requires urgent attention from the theoretical point of view. Indeed, the large increase of the blocking temperatures opens the possibility to incorporate molecular nanomagnets in devices operating at higher temperatures. But first, we need to deepen our understanding of the magnetic-behavior destroying process known as spin relaxation.

The target features that have commonly been addressed to block spin relaxation are (i) the ground electron spin quantum number *J* and (ii) the barrier height that separates the two components of the bistable ground state.^6^ Indeed, the search for new nanomagnets by increasing (i) and (ii) is consistent with an Orbach relaxation mechanism, which drives the spin population across the barrier. While this strategy has worked up to now,^7^,^8^ the recent interest in SIMs operating at high temperatures, where spin–vibration coupling dominates over relaxation, makes this scenario insufficient.^6^ Hence, to gain control of relaxation at increasing temperatures, spin–vibration coupling must be incorporated in the theoretical methods.

The current pursuit of predictive power is encouraging the development of fully *ab initio* methodologies.^9^,^10^ Nevertheless, first-principles evaluations of spin–vibration coupling are known to be computationally demanding.^3^,^9^,^10^ This fact constitutes a crucial limitation that makes state-of-the-art *ab initio* methods impractical when guiding efforts at the lab stage. Thus, searching for new methodologies able to circumvent this computational bottleneck is of paramount urgency. In the case of lanthanide-based SIMs, there already exist affordable semi-empirical approximations devoted to determining the electronic structure,^11^,^12^ whose accuracy can become comparable to that of *ab initio* calculations.^13^,^14^

Herein, we present an inexpensive first-principles method devoted to lanthanide and uranium SIMs, with the aim of evaluating vibration-induced spin relaxation. It allows estimating effective relaxation barriers *U*_eff_, relaxation pathways and relaxation times *τ* as a function of temperature. Crystal field parameters (CFPs) are determined by millisecond calculations, and only one CASSCF evaluation is required. The method identifies those vibrations promoting relaxation in order to re-design the given molecule and incorporates, for the first time, a temperature dependence in the spin–vibration coupling matrix elements. Contributions from spin–spin dipole coupling to *U*_eff_ and *τ* can be incorporated by resorting to recent first-principles models.^15^ Since the barrier height may increase from lanthanides to actinides due to a stronger ligand field, and given the challenging computational nature of the U^3+^ ion,^16^,^17^ we propose to evaluate the effectiveness of bis-metallocenium ligands on actinides and test the efficiency of our method on the hypothetical analog [U(Cp^ttt^)_2_]^+^ of [Dy(Cp^ttt^)_2_]^+^, which holds the latest record for the blocking temperature.^3^ To assess the validity in SIMs with ligands of a different nature, we also apply this method to the experimentally studied uranium-based SIM UTp_3_ (Tp^–^ = trispyrazolylborate) in order to rationalize its poor performance. The method consists of the following three steps.

## Methods

### Step 1

The relevant atom set is relaxed until reaching a minimum in its potential energy surface.^6^ This set may be the SIM itself^9^ or the unit cell of a crystal containing the SIM.^10^ After calculating the vibrational spectrum, we extract harmonic frequencies {*ν*_*j*_}_*j*_, reduced masses {*m*_*j*_}_*j*_, and displacement vectors 
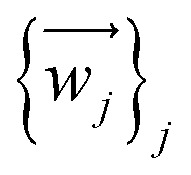
. These determine the 3D-direction in which each atom vibrates around its equilibrium position.

### Step 2

We perform a CASSCF calculation on the SIM experimental structure to extract the lowest 2*J* + 1 energies, where *J* is the metal ion ground electron spin quantum number. Then, once the coordinate origin is placed at the metal ion experimental position, the crystallographic coordinates of only the metal-coordinating atoms are introduced in the code SIMPRE; see the ESI.† 11,12 This code first calculates the CFPs by considering each coordinating atom to be an effective point charge and then performs a millisecond diagonalization of the ground *J* crystal field Hamiltonian. We apply the Radial Effective Charge (REC) model by varying the magnitude of the effective charges and their radial distance to the metal ion to fit the CASSCF energies;^18^,^19^ see the ESI.† Thus, we project the CASSCF information onto the first coordination sphere *via* effective REC parameters. Note that the contribution of the coordinating atoms to the ligand field almost recovers the whole effect of magnetic anisotropy. Nevertheless, one can include non-coordinating ligand atoms if a significant contribution is expected. To reduce computational costs, quite often it will be enough to maintain the same charge magnitude *Z*_*i*_ and radial distance contraction *D*_r_ in each coordinating atom.

This procedure is fully *ab initio*, but one can avoid the CASSCF evaluation and use the experimental energies if they are available. In this case, the experimental structure used in SIMPRE should be determined at the same temperature as that of the experimental energies. Now, the coordinating atom positions of the relaxed geometry are radially varied with the same fitting distance variations determined using SIMPRE. By using the same obtained charge values, SIMPRE calculates the equilibrium CFPs {(*A*_*k*_^*q*^〈*r*^*k*^〉))_eq_}_*k*,*q*_ in Stevens notation. Unlike lanthanides, excited states beyond the ground *J* multiplet may also influence the low-lying electronic structure of actinide SIMs. Thus, in the case of U^3+^, to determine the charge magnitude and the radial distance variation, the energy fitting must be replaced by a fitting of the SIMPRE CFPs to either the CASSCF or experimental CFPs. Yet, the energy fitting procedure can be maintained providing SIMPRE is replaced with the package CONDON at the stage when the energy set is determined for each set of CFPs calculated using SIMPRE from the varying *Z*_*i*_ and *D*_r_. CONDON contains the excited states beyond the ground *J* multiplet, and the CFPs must be introduced in Wybourne notation.^20^

The diagonalization in SIMPRE of the equilibrium crystal field Hamiltonian 
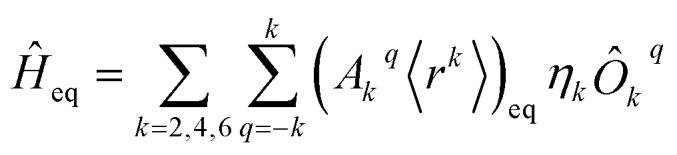
, where *η*_*k*_ is the Stevens coefficient and *Ô*_*k*_^*q*^ is the Stevens equivalent operator,^11^,^12^ provides the lowest 2*J* + 1 equilibrium eigenstates and energies; see the ESI.† For U^3+^, the diagonalization is performed in CONDON. The lowest 2*J* + 1 equilibrium eigenstates obtained using this code are truncated to the ordered basis set {|–*J*〉, …, |+, …, |+*J*〉} of the ground } of the ground *J* multiplet and then renormalized.

The perturbed Hamiltonians 
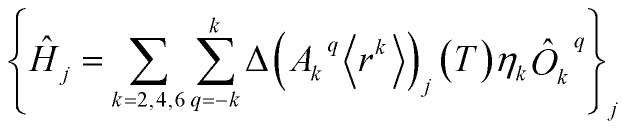
, which are also built on the above ordered basis set, account for the perturbation to the equilibrium electronic structure from each vibrational mode *j*; see the ESI.† Their determination requires the estimation of the temperature-dependent change Δ(*A*_*k*_^*q*^〈*r*^*k*^〉))_*j*_(*T*) produced in (*A*_*k*_^*q*^〈*r*^*k*^〉))_eq_ after activating each mode *j*. We use a model derived by us elsewhere,^9^ which provides the following perturbative expression up to the second-order in mode coordinate *Q*_*j*_:1
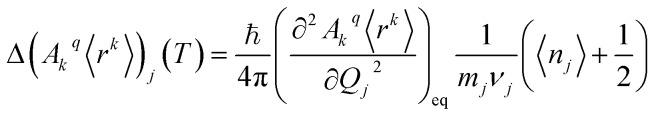



Thus, each *Ĥ*_*j*_ allows determining the spin–vibration coupling matrix element allows determining the spin–vibration coupling matrix element 〈*i*|*Ĥ*_*j*_|*f*〉; see the ESI.; see the ESI.† Temperature dependence is introduced for the first time in these elements through each boson number Temperature dependence is introduced for the first time in these elements through each boson number 〈*n*_*j*_〉 = 1/(e = 1/(e^*hν*_*j*_/*k*_B_*T*^ – 1). The procedure to calculate the derivatives (∂^2^*A*_*k*_^*q*^〈*r*^*k*^〉/∂/∂*Q*_*j*_^2^)_eq_ can be found in the ESI.†

In any case, proper tuning of the chemical structure aimed at reducing the variations in Δ(*A*_*k*_^*q*^〈*r*^*k*^〉))_*j*_ would improve the molecular nanomagnet performance.^9^ Indeed, the aforementioned reduction would make the matrix elements Indeed, the aforementioned reduction would make the matrix elements 〈*i*|*Ĥ*_*j*_|*f*〉 and the transition rate and the transition rate *γ* smaller, since the perturbed Hamiltonians *Ĥ*_*j*_ are proportional to Δ(*A*_*k*_^*q*^〈*r*^*k*^〉))_*j*_. For instance, this could be undertaken by increasing the harmonic frequencies *ν*_*j*_ of the relevant vibrational modes with proper ligand modification.

Of course, a legitimate question is whether these inexpensive calculations produce qualitatively similar CFPs at the equilibrium and distorted geometries as compared to the ones derived from *ab initio* calculations. This is what has been confirmed in a very recent study on the dysprosium-based SIM Dy-5*,^21^ which holds the latest record for the blocking temperature.

### Step 3

This step is undertaken by solving the master equation, eqn (2),^3^,^22^,^23^ which describes the time evolution of the spin population across the lowest 2*J* + 1 equilibrium eigenstates. The energy that induces the spin to relax comes from the coupling with surrounding vibrations. Intuitively, at each time *t* there is a probability *p*_*i*_(*t*) of being in an eigenstate |*i*〉. At a time . At a time *t* + d*t* the system may make a transition to a different eigenstate |*f*〉 with a probability with a probability *γ*_*if*_d*t*, either by absorbing or by emitting a vibration quantum. The net difference between the incoming *γ*_*fi*_*p*_*i*_ and outgoing *γ*_*if*_*p*_*f*_ spin populations equals the change in time of *p*_*i*_. Thus, once the transitions are assumed to be independent, these probabilities evolve as follows:2
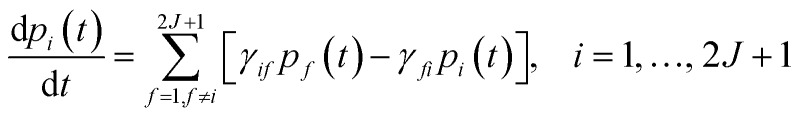



The transition rates *γ*_*if*_ and *γ*_*fi*_ account for the spin population flow between |*i*〉 and | and |*f*〉, and their expressions depend on the relaxation process to model. We include two important processes: (i) Orbach and (ii) second-order Raman relaxation; see the ESI., and their expressions depend on the relaxation process to model. We include two important processes: (i) Orbach and (ii) second-order Raman relaxation; see the ESI.† The determination of the most likely relaxation pathway provides further insight into relaxation. This allows identifying the vibrations that promote each relaxation step, and modifications on the molecular structure can then be proposed to suppress relaxation. All details are found in the ESI.† 3,22–24 The same procedure applies if the crystal field Hamiltonians are expanded to include a static magnetic field *via* a Zeeman term.

## Two case studies: UTp_3_ and [U(Cp^ttt^)_2_]^+^

A handful of actinide complexes, mostly based on U^3+^, have been reported over the past few years as SIMs, although mostly with a poor magnetic behavior.^25^ We choose to apply our theoretical approach to two complementary case studies. In the first place, we studied UTp_3_, Tp^–^ = trispyrazolylborate, where the magnetic ion is directly bonded to nine pyrazole rings in a nearly exact *D*_3h_ tricapped trigonal prism coordination environment.^26^ This is one of the few known uranium SIMs for which spectroscopic characterization has been performed and which thus can serve to validate our calculation of energy levels and wave-functions in these types of challenging systems. Secondly, we studied [U(Cp^ttt^)_2_]^+^, a hypothetical molecule that is analogous to the dysprosocenium SIM [Dy(Cp^ttt^)_2_]^+^. In this system, the *f*-ion is sandwiched between two *tert*-butyl(cyclopentadienyl) (Cp^ttt^) ligands, and this gives rise to an overall linear coordination geometry that is slightly bent. Recently, a uranium-based SIM with a metallocenium-like ligand set similar to Cp^ttt^ has been synthesized and experimentally characterized. This demonstrates the chemical viability of extending this particular family of ligands to uranium.^27^

**Fig. 1:**
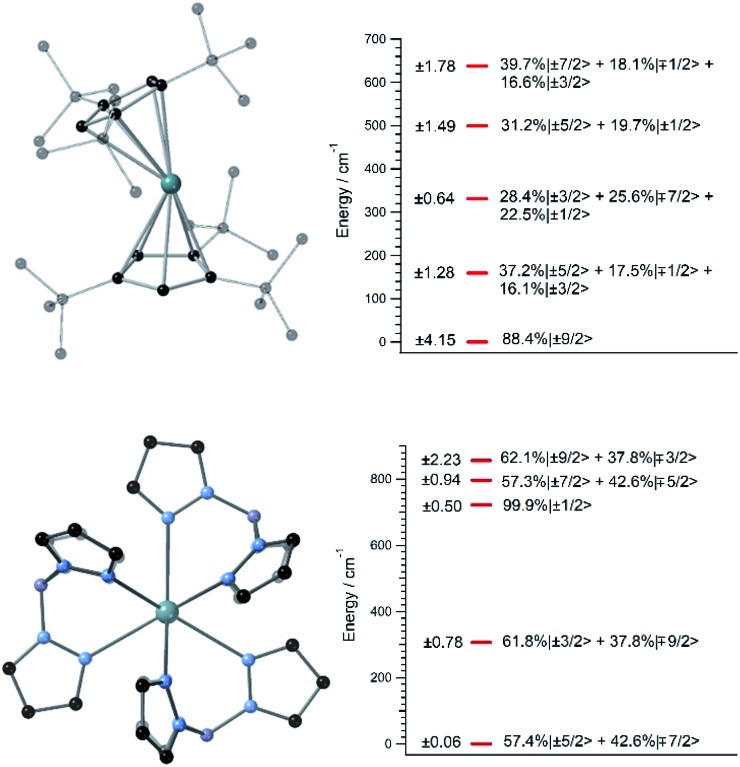
(Left top and bottom) [U(Cp^ttt^)_2_]^+^ and UTp_3_ relaxed geometries. Hydrogen atoms are omitted for clarity. Green: U, black: C, blue: N, violet: B. (Right top and bottom) [U(Cp^ttt^)_2_]^+^ and UTp_3_ lowest 2*J* + 1 = 10 equilibrium energies with wave-functions on the right (amplitudes < 15% are not shown) and their *J*_*z*_ operator expectation values on the left. The wave-functions are written in terms of the ground *J* = 9/2 multiplet of U^3+^.

Since we lack the experimental structures of [U(Cp^ttt^)_2_]^+^ and UTp_3_, we use the ones of [Dy(Cp^ttt^)_2_]^+^ and NdTp_3_ instead.^3^,^26^ By replacing the corresponding lanthanide ion with U^3+^, we carry out geometry relaxation and vibrational spectrum calculation in both systems; see Fig. 1 and the ESI.† Now, we perform a CASSCF evaluation on real structures of [U(Cp^ttt^)_2_]^+^ and UTp_3_ to obtain {(*A*_*k*_^*q*^〈*r*^*k*^〉))_eq_}_*k*,*q*_. In the case of [U(Cp^ttt^)_2_]^+^, this is not possible and we will proceed as explained in the ESI† to determine the equilibrium electronic structure; see Fig. 1. Concerning UTp_3_, we will use the spectroscopic energy scheme to obtain its equilibrium electronic structure; see also the ESI† and Fig. 1.

In contrast with that of [U(Cp^ttt^)_2_]^+^, the energy level scheme of UTp_3_, see Fig. 1, is rather typical when compared with those of other studied uranium complexes.^17^ The ground doublet of UTp_3_ presents a heavy mixing between the |*m*_*J*_〉 components ±5/2 and ∓7/2, and this results in an almost perfect cancellation of the expectation value 〈 components ±5/2 and ∓7/2, and this results in an almost perfect cancellation of the expectation value 〉 components ±5/2 and ∓7/2, and this results in an almost perfect cancellation of the expectation value 〈*J*_*z*_〉 = ±0.06. This seems to be a generalized feature that results from the combination of: (a) a non-perfect axial coordination geometry, where the ±5/2 and ∓7/2 components are favoured to the detriment of the maximum values ±9/2, and (b) the = ±0.06. This seems to be a generalized feature that results from the combination of: (a) a non-perfect axial coordination geometry, where the ±5/2 and ∓7/2 components are favoured to the detriment of the maximum values ±9/2, and (b) the *D*_3h_ coordination symmetry, which results in a heavy mixing of levels differing by Δ*m*_*J*_ = 6 (this is precisely the case for ±5/2 and ∓7/2). On the other hand, [U(Cp^ttt^)_2_]^+^ also displays noticeable mixing as a consequence of the *C*_1_ symmetry, although in this case the axial ligand distribution stabilizes a ground doublet mainly characterized by the component ±9/2 with a weight around 80%. This difference in the electronic structure already means that [U(Cp^ttt^)_2_]^+^ should present a better prospect for slow relaxation of the magnetization as compared with UTp_3_.

**Fig. 2:**
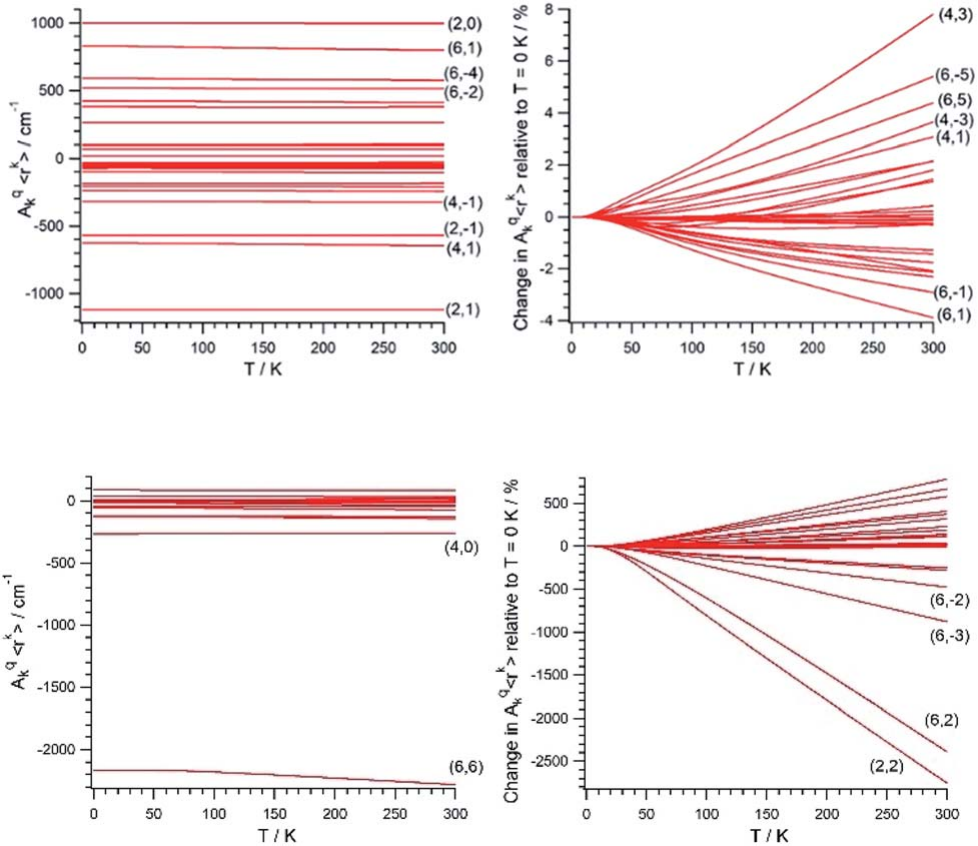
Absolute (left) and relative to *T* = 0 K (right) thermal evolution of the CFPs of [U(Cp^ttt^)_2_]^+^ (top) and UTp_3_ (bottom). Some CFPs are identified as (*k*,*q*), where *k* and *q* are the scripts *k* = 2, 4, 6 and *q* = –*k*, …, +*k*.

Once (*A*_*k*_^*q*^〈*r*^*k*^〉))_eq_ and (∂^2^*A*_*k*_^*q*^〈*r*^*k*^〉/∂/∂*Q*_*j*_^2^)_eq_ are determined, we calculate the CFP thermal evolution.^9^ From Fig. 2, important contributions from off-diagonal CFPs are observed for both systems. Importantly, one has to note the clearly dominant contribution of *B*_6_^6^, which governs the crystal field splitting in UTp_3_. This fact opposes good SIM behavior, where the diagonal CFPs should largely dominate over the off-diagonal ones. However, a striking difference between UTp_3_ and [U(Cp^ttt^)_2_]^+^ can be seen from their CFP thermal evolution. Indeed, the CFPs of [U(Cp^ttt^)_2_]^+^ are almost constant, with variations of up to a few percentage points at most. In contrast, there exists a marked thermal dependence in the case of UTp_3_, where the relative variations are even two orders of magnitude larger.

Simple symmetry arguments can be used to give some intuitive meaning to these numerical results. In the case of [U(Cp^ttt^)_2_]^+^, the molecular symmetry is not ideal, meaning no CFPs cancel for symmetry reasons. As a consequence, there are no significant changes caused to geometrical distortions of any reasonable size. This, added to the molecular rigidity that is characteristic of metallocenium complexes, results in a relative insensibility to thermal effects. Since the molecular geometry in this case is quite axial, the *B*_2_^0^ parameter remains dominant at all temperatures. The opposite situation happens for UTp_3_, where the perfect *D*_3h_ symmetry means many CFPs are cancelled at the equilibrium geometry. Thus, even moderate geometrical distortions cause dramatic relative changes in many CFPs, as indeed is the result of the calculations. In the special case of *B*_6_^6^, this CFP is governed by a spherical harmonic of *D*_6h_ symmetry in such a way that all 9 donor atoms contribute with the same sign. Because the molecule presents an overall *C*_3_ symmetry, certain concerted geometrical distortions that result from molecular vibrations couple with an unusually strong strength with this CFP: *B*_6_^6^ is at the same time the dominant parameter in the crystal field Hamiltonian and the one with the largest absolute thermal effect. All in all, the dynamical effects coincide with the static picture in discarding the potential as a SIM of UTp_3_ in particular, and possibly of *C*_3_-type U^3+^ complexes in general.

In order to illustrate step 3 of the methodology we will now focus on the most promising case, namely [U(Cp^ttt^)_2_]^+^. Unfortunately, to the best of our knowledge, the uranium-based molecular nanomagnets reported so far in the literature – including UTp_3_ – do not exhibit slow relaxation of the magnetization above *ca.* 5 K. This prevents us from applying step 3 to UTp_3_, since around and below this temperature a very high numerical noise leads to poorly reliable results; see the ESI.†

**Fig. 3:**
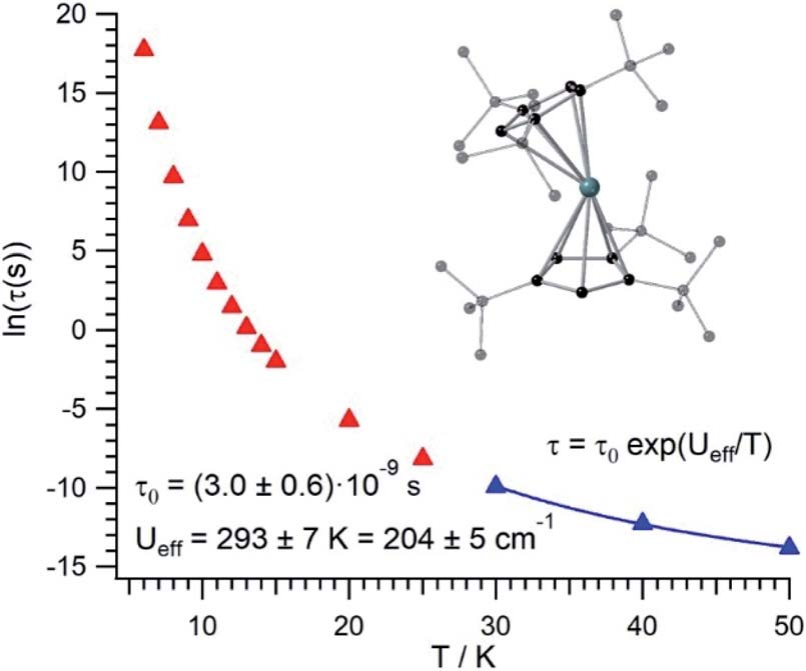
[U(Cp^ttt^)_2_]^+^ thermal evolution of the Orbach-based relaxation time *τ*, along with fit to determine both the Orbach prefactor *τ*_0_ and the effective barrier *U*_eff_ in the thermally activated regime (*T* ≥ 30 K).

**Fig. 4:**
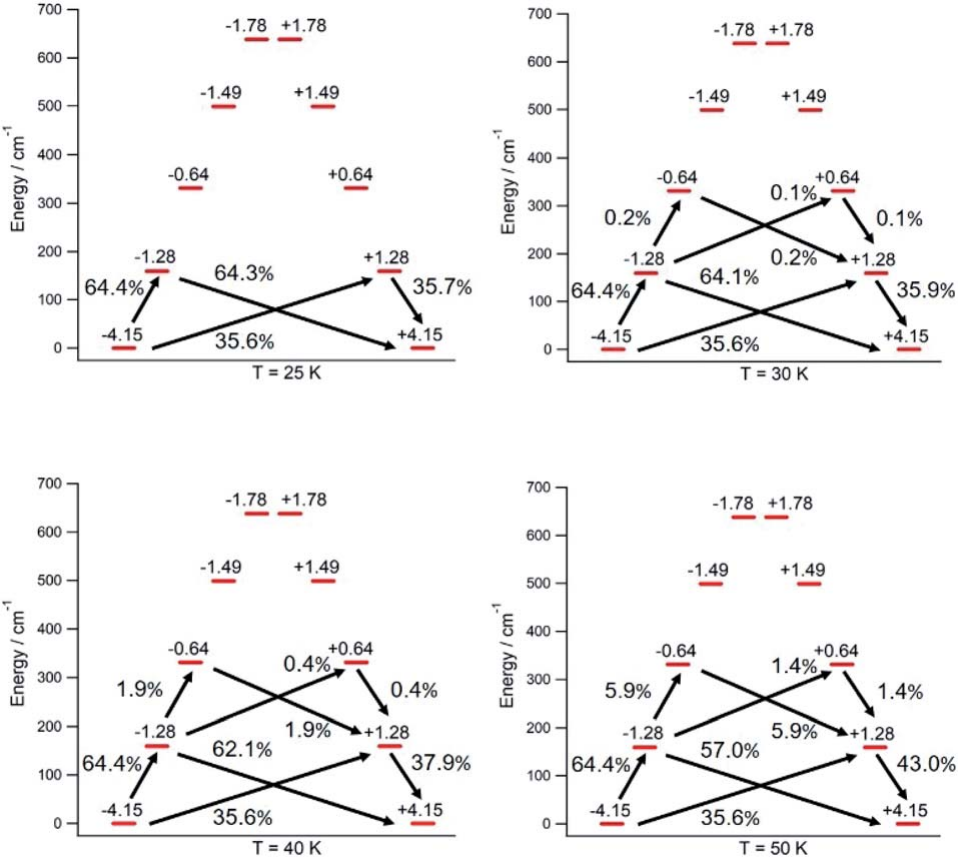
[U(Cp^ttt^)_2_]^+^ Orbach-driven relaxation pathways starting at the Orbach-driven relaxation pathways starting at the 〈*J*_*z*_〉 = −4.15 equilibrium eigenstate with a unit population. The outgoing population sum from a given eigenstate equals the incoming population sum to the same eigenstate. Transient populations less than 0.1% are not shown. = –4.15 equilibrium eigenstate with a unit population. The outgoing population sum from a given eigenstate equals the incoming population sum to the same eigenstate. Transient populations less than 0.1% are not shown.

The thermal dependence of the relaxation time *τ* when the Orbach transition rates are used in eqn (2) is shown in Fig. 3. Above 30 K, where there exist a high enough number of available phonons, the thermally activated regime is at play and the spin population crosses the barrier through excited doublets; Fig. 4. Below 30 K, little or rather negligible spin population is promoted to the second excited doublet, which mostly tunnels the barrier through the first excited doublet. Nevertheless, let us recall that as the temperature is decreased other mechanisms could come into play and even dominate over the Orbach-based one such as quantum tunneling between the ground doublet components. This process is not considered by our approach but commonly observed at low temperatures.^4^,^5^

In this thermally activated regime, the estimated effective barrier *U*_eff_ = 293 K is in the range of hundreds of kelvin, which is normal in a large set of molecular nanomagnets,^28^ and is found around 40 cm^–1^ above the first excited doublet in Fig. 1. The Orbach prefactor, *τ*_0_ = e^–19.62^ = 3.0 × 10^–9^ s, is within the usual range (10^–6^–10^–10^ s) for SIMs with a barrier of a comparable height. We also evaluated eqn (2) with the second-order Raman transition rates. The Raman-based *τ* values, see Table S1,† are much larger than the ones in Fig. 3. Thus, the second-order Raman process should be discarded as a competitive mechanism in the experiment, as found in [Dy(Cp^ttt^)_2_]^+^.^3^

Even employing bis-metallocenium ligands, known to offer a strong axial crystal field in Dy-based SIMs,^3^–^5^ our calculated [U(Cp^ttt^)_2_]^+^ effective barrier (293 K) is one order of magnitude below those reported for [Dy(Cp^ttt^)_2_]^+^ (1760 K)^3^ and Dy-5* (2217 K).^5^ Besides, the maximum temperature in [U(Cp^ttt^)_2_]^+^ (∼40–50 K) at which the experimental relaxation time is still above the standard experimental detection limit (10^–5^–10^–6^ s) is also clearly smaller compared to that in [Dy(Cp^ttt^)_2_]^+^ (∼112 K)^3^ and Dy-5* (∼138 K).^5^ As can be expected for a decrease of about one order of magnitude in the barrier height, the calculated [U(Cp^ttt^)_2_]^+^ Orbach prefactor *τ*_0_ = 3.0 × 10^–9^ s is two to three orders of magnitude above the ones corresponding to the two Dy-based SIMs (*τ*_0_ ∼ 2.0 × 10^–11^ s and *τ*_0_ ∼ 4.2 × 10^–12^ s, resp.).^3^,^5^

Our [U(Cp^ttt^)_2_]^+^ Orbach prefactor is among the smallest ones that have been experimentally determined in uranium SIMs.^16^,^17^ On the other hand, there do exist two significant advances with respect to previous uranium SIMs: (i) the standard effective barrier is in the range of dozens of kelvin,^16^,^17^ while that for [U(Cp^ttt^)_2_]^+^ reaches several hundreds of kelvin (∼293 K); (ii) by assuming that the thermally activated regime dominates between 30 K and 50 K in [U(Cp^ttt^)_2_]^+^, while it is not possible to determine relaxation times beyond ∼5 K,^16^,^17^ the experimental detection limit *τ* ∼ 10^–5^–10^–6^ s in the case of [U(Cp^ttt^)_2_]^+^ would be found at 40–50 K. Thus, it seems that [U(Cp^ttt^)_2_]^+^ is not expected to display hysteresis temperatures that compete with the ones of [Dy(Cp^ttt^)_2_]^+^ and Dy-5* SIMs. For instance, in a typical hysteresis loop swept at 2 mT s^–1^ between –1 T and +1 T, which means to store classical information for 2000 s, a blocking temperature below 9 K would be observed in [U(Cp^ttt^)_2_]^+^ according to Fig. 3. This is unsurprising since, after all, the Cp^ttt^ rings were optimized for dysprosium and may present somewhat different requirements in uranium. However, our methodology is efficient enough to offer a path forward in the rational design of ligands that result in uranium SIMs with optimized performance.

Let us now analyze the [U(Cp^ttt^)_2_]^+^ vibrations determining the transition rates that drive the relaxation pathways in Fig. 4, see the animations and the ESI,† and offer some strategies to reduce their detrimental effects. Two of them involve methyl rotations in the Cp^ttt^ ring substituents. These rotations could be partially suppressed by replacing the methyl groups (–CH_3_) with the heavier fluorinated analogs –CF_3_. A quick inspection shows that there exist similar vibrations promoting relaxation in [Dy(Cp^ttt^)_2_]^+^, Dy-5* and [U(Cp^ttt^)_2_]^+^. On one hand, rocking deformations of the Cp^ttt^ rings where directly bonded hydrogen atoms move towards and away from the metal ion are present in [Dy(Cp^ttt^)_2_]^+^ and [U(Cp^ttt^)_2_]^+^. On the other hand, breathing vibrations where the two coordinating rings move towards and away from the metal ion are found in [U(Cp^ttt^)_2_]^+^ and Dy-5*. The rocking deformations in [Dy(Cp^ttt^)_2_]^+^, also found in [U(Cp^ttt^)_2_]^+^, were already blocked in ref. 5 by placing bulkier substituents in the coordinating rings. It worked as expected since both the effective barrier and the blocking temperature were increased with respect to those of [Dy(Cp^ttt^)_2_]^+^. A possible strategy which has not yet been explored to remove the breathing vibrations could be bonding the two coordinating rings, such as in stapled bis-phthalocyanines. Moreover, the frequencies of the [U(Cp^ttt^)_2_]^+^ detrimental vibrations, see the ESI,† closely match the gaps between the equilibrium ground and first excited doublets (159.3 cm^–1^) and the first and second excited doublets (171.7 cm^–1^); Fig. 1. Thus, the [U(Cp^ttt^)_2_]^+^ performance would also benefit from any structural modification that takes these frequencies out of resonance with respect to these gaps.

## Conclusions

All in all, the most important conclusions of this work are the following. We have proposed a novel first-principles methodology aimed to simulate vibration-induced spin relaxation in *f*-block SIMs. The method offers the important advantage of drastically reducing the computation time while keeping the calculation accuracy within an acceptable range. Indeed, all but one of the expensive CASSCF calculations required in previous methods are replaced by millisecond calculations. Besides, our approach introduces for the first time a temperature dependence in the spin–vibration coupling matrix elements. To demonstrate this methodology, we consider two case studies. First, we revisit UTp_3_, a uranium SIM that has been studied spectroscopically and therefore allows us to apply our methodology with the highest-quality data. Here we find a heavy mixing in the ground doublet which results in an almost perfect cancellation of the expectation value , a uranium SIM that has been studied spectroscopically and therefore allows us to apply our methodology with the highest-quality data. Here we find a heavy mixing in the ground doublet which results in an almost perfect cancellation of the expectation value 〈*J*_*z*_〉. Furthermore, the intense thermal dependence of the CFPs evidences a strong coupling between molecular vibrations and the spin energy levels. This means that both from the static and from the dynamic point of view UTp. Furthermore, the intense thermal dependence of the CFPs evidences a strong coupling between molecular vibrations and the spin energy levels. This means that both from the static and from the dynamic point of view UTp_3_ is expected to display poor SMM behavior. In the second place, we study the high-performing SIM [Dy(Cp^ttt^)_2_]^+^ and find that the replacement of Dy^3+^ with U^3+^ does not result in an enhanced performance. Yet, [U(Cp^ttt^)_2_]^+^ does seem to outperform all previously reported uranium SIMs. One of the critical factors that promote spin relaxation in [U(Cp^ttt^)_2_]^+^ is the noticeable mixing among the |*m*_*J*_〉 components in the equilibrium eigenstates. Importantly, this mixing is also found in previously reported uranium SIMs, components in the equilibrium eigenstates. Importantly, this mixing is also found in previously reported uranium SIMs,^16^,^17^,^29^ but not in the cutting-edge Dy-based SIMs [Dy(Cp^ttt^)_2_]^+^ and Dy-5* even though the ligand coordination is not strictly axial.^3^,^5^ Among those vibrations that promote spin relaxation, there are still atomic movements left to block. These involve methyl rotations and breathing deformations, which could be removed by fluorination and stapling the coordinating rings to each other, respectively. Hence, we conclude that there may still be room for further improvement in these bis-metallocenium-based uranium SIMs.

## Conflicts of interest

There are no conflicts to declare.

## Supplementary Material

Supplementary information

Supplementary information

Supplementary information

Supplementary information

## References

[cit1] Sessoli R., Gatteschi D., Caneschi A., Novak M. A. (1993). Nature.

[cit2] Ishikawa N., Sugita M., Ishikawa T., Koshihara S.-y., Kaizu Y. (2004). J. Phys. Chem. B.

[cit3] Goodwin C. A. P., Ortu F., Reta D., Chilton N. F., Mills D. P. (2017). Nature.

[cit4] McClain K. R., Gould C. A., Chakarawet K., Teat S. J., Groshens T. J., Long J. R., Harvey B. G. (2018). Chem. Sci..

[cit5] Guo F.-S., Day B. M., Chen Y.-C., Tong M.-L., Mansikkamäki A., Layfield R. A. (2018). Science.

[cit6] Escalera-Moreno L., Baldoví J. J., Gaita-Ariño A., Coronado E. (2018). Chem. Sci..

[cit7] Baldoví J. J., Cardona-Serra S., Clemente-Juan J. M., Coronado E., Gaita-Ariño A., Palii A. (2012). Inorg. Chem..

[cit8] Rinehart J. D., Long J. R. (2011). Chem. Sci..

[cit9] Escalera-Moreno L., Suaud N., Gaita-Ariño A., Coronado E. (2017). J. Phys. Chem. Lett..

[cit10] Lunghi A., Totti F., Sessoli R., Sanvito S. (2017). Nat. Commun..

[cit11] Baldoví J. J., Clemente-Juan J. M., Coronado E., Gaita-Ariño A., Palii A. (2014). J. Comput. Chem..

[cit12] Cardona-Serra S., Escalera-Moreno L., Baldoví J. J., Gaita-Ariño A., Clemente-Juan J. M., Coronado E. (2016). J. Comput. Chem..

[cit13] Hallmen P. P., Köppl C., Rauhut G., Stoll H., van Slageren J. (2017). J. Chem. Phys..

[cit14] Calvello S., Piccardo M., Rao S. V., Soncini A. (2017). J. Comput. Chem..

[cit15] Aravena D. (2018). J. Phys. Chem. Lett..

[cit16] Coutinho J. T., Perfetti M., Baldoví J. J., Antunes M. A., Hallmen P. P., Bamberger H., Crassee I., Orlita M., Almeida M., van Slageren J., Pereira L. C. J. (2019). Chem.–Eur. J..

[cit17] Baldoví J. J., Cardona-Serra S., Clemente-Juan J. M., Coronado E., Gaita-Ariño A. (2013). Chem. Sci..

[cit18] Baldoví J. J., Borrás-Almenar J. J., Clemente-Juan J. M., Coronado E., Gaita-Ariño A. (2012). Dalton Trans..

[cit19] Baldoví J. J., Gaita-Ariño A., Coronado E. (2015). Dalton Trans..

[cit20] van Leusen J., Speldrich M., Schilder H., Kögerler P. (2015). Coord. Chem. Rev..

[cit21] Ullah A., Cerdá J., Baldoví J. J., Varganov S. A., Aragó J., Gaita-Ariño A. (2019). J. Phys. Chem. Lett..

[cit22] Gatteschi D., Sessoli R. and Villain J., Molecular Nanomagnets, Oxford University Press, Oxford, 2006.

[cit23] Alexander M. H., Hall G. E., Dagdigian P. J. (2011). J. Chem. Educ..

[cit24] Orbach R. (1961). Proc. - R. Soc. Edinburgh, Sect. A: Math. Phys. Sci..

[cit25] Escalera-Moreno L., Baldoví J. J., Gaita-Ariño A., Coronado E. (2019). Inorg. Chem..

[cit26] Rinehart J. D., Long J. R. (2012). Dalton Trans..

[cit27] Guo F.-S., Chen Y.-C., Tong M.-L., Mansikkamäki A., Layfield R. A. (2019). Angew. Chem..

[cit28] Yang J.-W., Tian Y.-M., Tao J., Chen P., Li H.-F., Zhang Y.-Q., Yan P. F., Sun W.-B. (2018). Inorg. Chem..

[cit29] Antunes M. A., Coutinho J. T., Santos I. C., Marçalo J., Almeida M., Baldoví J. J., Pereira L. C. J., Gaita-Ariño A., Coronado E. (2015). Chem.–Eur. J..

