# Supplementary material for: Design of high-temperature f-block molecular nanomagnets through the control of vibration-induced spin relaxation
Source: Chem Sci. 2019 Dec 2;11(6):1593–8. doi: 10.1039/c9sc03133b (PMC7025469; doi:10.1039/c9sc03133b)
Supplement: Supplementary file 1 [file SC-011-C9SC03133B-s001.pdf]

Electronic Supplementary Material (ESI) for Chemical Science.
This journal is © The Royal Society of Chemistry 2020

Supplementary Information

Design of high-temperature f-block molecular nanomagnets through the control of vibration-induced spin relaxation

L. Escalera-Moreno,[a] José J. Baldoví,[*,a] A. Gaita-Ariño, E. Coronado[a]

a. Instituto de Ciencia Molecular (ICMol), Universidad de Valencia, c/ Catedrático José Beltrán 2, 46980, Paterna, Spain

*. Corresponding author

Geometry relaxation and vibrational spectrum calculation

This step is commonly accomplished by methods based on Density Functional Theory, as they provide an acceptable accuracy-cost ratio. We use the package Gaussian09 to perform the geometry relaxation and the vibrational spectrum calculation.[1] In the folder Relax-Geom-Vib-Spec-Calc one finds the input uranocenium.inp and the output uranocenium.log which crashed and was subsequently restarted in uranocenium-r.log. In the output uranocenium-r.log one finds the relaxed geometry, the harmonic frequencies, the reduced masses, the force constants and the displacement vectors of each vibrational mode.

In the input uranocenium.inp one finds the experimental crystallographic geometry of $[Dy(Cp^{ttt})_2]^+$ (ref. 3 in main text), where we have replaced Dy^{3+} by U^{3+}. The resultant structure has a charge of +1 and a ground spin multiplicity (2S+1) of 4. This structure is relaxed in vacuum. Of course, a more realistic relaxation often requires to include the nearest environment of the molecule, although it may be kept frozen in order not to increase the computational cost (ref. 9 in main text). Since our main goal in this manuscript is to focus on the novel methodology that we are proposing, we decided to relax this geometry in vacuum in order not to unnecessarily increase the computational cost. Thus, we apply our method to $[U(Cp^{ttt})_2]^+$ and study its magnetic relaxation mediated only by molecular vibrations in vacuum. As a matter of fact, the experimental geometry of $[Dy(Cp^{ttt})_2]^+$ was also relaxed in vacuum elsewhere by independent authors, and reasonable results regarding geometry relaxation, vibrational spectrum and relaxation dynamics were obtained (ref. 3 in main text). We employ a similar procedure as in $[Dy(Cp^{ttt})_2]^+$, namely, the functional was PBEPBE, complemented with the GD3BJ empirical dispersion. Basis sets: cc-pVDZ for carbon and hydrogen atoms, and the effective core potential Stuttgart-RSC-1997-ECP for the uranium atom. The same description and procedure applies to UTp_3.

Generation of distorted geometries. Determination of the CFP second derivatives at the relaxed geometry.

From the vibrational spectrum calculation (see output uranocenium-r.log), we get a $3N$-dimensional Cartesian displacement vector $\vec{w}_j$ for each vibrational mode $j = 1,...,R$, where N is the number of vibrating atoms and R is the number of vibrational modes. Given the $3N$-dimensional Cartesian vector $\vec{v}_{eq}$ that contains the atom coordinates of the relaxed geometry, each distorted geometry d, represented by the $3N$-dimensional Cartesian vector $\vec{v}_j^d$, is generated as $\vec{v}_j^d = \vec{v}_{eq} + Q_j^d \vec{w}_j$, where Q_j^d is a given real value of the distortion coordinate Q_j of the vibrational mode j. Note that when $Q_j^d = 0$, the relaxed geometry is recovered $\vec{v}_j^d = \vec{v}_{eq}$. We are going to explain now how we choose the set of values $\{Q_j^d\}_d$ for a given vibrational mode j. First, we calculate the minimum value $Q_j^{\min}$, i.e., the minimum displacement of the given vibrational mode j. The key idea is to produce a significant distortion respect to the relaxed geometry. In other words, the distortion must produce on each component of each vibrating atom position a change at least equal to the experimental crystallographic error of this component. Since each displacement vector is normalized, we have $Q_j^{\min} = \left\| \vec{v}_j^{\min} - \vec{v}_{eq} \right\|$, i.e., $Q_j^{\min} = \sqrt{\sum_{i=1}^{N} \sum_{\alpha=x,y,z} \left(\alpha_j^{i,\min} - \alpha_{eq}^i\right)^2}$, where $\left(x_{eq}^i, y_{eq}^i, z_{eq}^i\right)$ is the equilibrium position of the atom i, and $\left(x_j^{i,\min}, y_j^{i,\min}, z_j^{i,\min}\right)$ is the minimum distorted position of the atom i under the vibrational mode j. As said, each $\alpha_j^{i,\min} - \alpha_{eq}^i$ must equal $\delta\alpha^i$, which is the experimental crystallographic error in the α component of the atom i. Thus, we propose that the minimum displacement $Q_j^{\min}$ for the given vibrational mode j be: $Q_j^{\min} = \sqrt{\sum_{i=1}^{N} \sum_{\alpha=x,y,z} \left(\delta\alpha^i\right)^2}$. Standard X-ray crystallographic techniques do not usually detect hydrogen atoms because of their low electron density, and the relevant post-processing software does not attribute any crystallographic error to the hydrogen atoms in the experimental structure. Thus, we decide not to include any hydrogen atom in the expression of $Q_j^{\min}$. Sometimes, there exist components in the displacement vectors $\vec{w}_j$ that are zero or close enough to zero. This means that some components of some atoms do not change or hardly change from their equilibrium values when the vibrational mode j is working. We also decide to exclude from the $Q_j^{\min}$ expression those atom components which do not change or hardly change. To decide which atom components must be excluded, we use a threshold in the corresponding component of the displacement vector. If the absolute value of the corresponding component in the displacement vector is not greater than the threshold, then we set $\delta\alpha^i = 0$, see below.

First, to determine the minimum displacements $Q_j^{\min}$ we include the code prefactor-v2.f90 in the folder Home-Made-Codes, along with the input prefactor-v2-input and the output prefactor-v2-output. Inside the code, one finds the variables nmod (number of vibrational modes), nato (number of atoms, namely, all vibrating atoms but the hydrogen atoms), and the threshold th

to decide whether a given component of a given displacement vector is small enough or not. In the input prefactor-v2-input, one first needs to introduce the three lattice parameters (in Angstroms) a , b , c of the experimental structure. Since we lack an experimental structure of $[U(Cp^{ttt})_2]^+$, we used the lattice parameters of $[Dy(Cp^{ttt})_2]^+$. Note that the protocol to choose the Q_j^d values for each vibrational mode j is not unique, we only need some distorted geometries around the relaxed geometry in order to calculate the CFPs second derivatives respect to the distortion coordinate for each vibrational mode j . Thus, it is not strictly necessary to have some of the real parameters of the system under study. We need the lattice parameters (in Angstroms) because in prefactor-v2-input we introduce the crystallographic errors in fractional coordinates, and $Q_j^{\min}$ must be calculated in Angstroms. Whenever the crystallographic errors are introduced in orthogonal coordinates in Angstroms, one will set the three lattice parameters to be 1.0. After the lattice parameters in prefactor-v2-input, we introduce the crystallographic errors in fractional coordinates as said. The first row is for the metal, and the following rows are for the remaining atoms, in this case, carbon atoms. These crystallographic errors are from the $[Dy(Cp^{ttt})_2]^+$ experimental structure. The error in the x-component is more or less the same for all the carbon atoms. Thus, we used the average value for the x-component of the error in each carbon atom. We analogously proceed for the y and z components of the error. To end up, after the crystallographic errors, the displacement vectors are introduced as directly provided by the output of Gaussian09 (keep the blank lines between lattice parameters, crystallographic errors and displacement vectors). From these displacement vectors, we must to remove those rows corresponding to the hydrogen atoms whenever they exist. The order both in the crystallographic error rows and in the displacement vectors rows has to be the same. For example, since the first row in each one of our displacement vectors corresponds to the metal atom, the first row of the crystallographic errors table must also correspond to the metal atom. Since we gave the same crystallographic error to each carbon atom and since all remaining rows of the displacement vectors (once those rows corresponding to hydrogen atoms have been removed) correspond to carbon atoms, there is no need to care about the order of the remaining rows in the crystallographic errors table. The lapack library is not required. To run the code, we use the compiler gfortran and the command line: gfortran –o aa prefactor-v2.f90 (aa is the executable name). In the output prefactor-v2-output, each minimum displacement $Q_j^{\min}$ is given after printing each displacement vector as “minimum displacement (Angstroms)”.

Second, we calculate how many distorted geometries we need for each vibrational mode j . For that purpose, we include the excel file Number-Distorted-Geom in the folder Home-Made-Codes. In this file, the input consists of the harmonic frequencies ν and the force constants k as directly provided by the Gaussian09 output uranocenium-r.log. If the values of ν and k are changed, the values of l.c.(n=0) and l.c.(n=1) will be automatically changed. The variables l.c.(n=0) and l.c.(n=1) are the classical limits (Angstroms) in the distortion coordinate corresponding to the ground and first excited harmonic vibrational levels of a given vibrational mode j . Let us recall that the minimum displacement $Q_j^{\min}$ (Angstroms) for each vibrational mode j is provided by the code prefactor-v2.f90. We take the maximum displacement (Angstroms) of the distortion coordinate of a given vibrational mode j as the value $Q_j^{\min} \cdot s_j$, where s_j is the natural number such that $Q_j^{\min} \cdot s_j$ is the smallest real number above l.c. (n=0). Thus, the number of distorted geometries for the given vibrational mode j is $2 \cdot s_j$.

To end up, we need to generate the distorted geometries for each vibrational mode. For that, we include the code geom_dist.f90 in the folder Home-Made-Codes. In the input geom_dist_input, we first provide the minimum displacement $Q_j^{\min}$ (Angstroms) and the natural number s_j for each vibrational mode j. These pairs -each one of them corresponds to a given vibrational mode j - have to be ordered with increasing frequency. Then (keep the blank line), we write the relaxed geometry in Cartesian coordinates (Angstroms) along with the chemical symbol of each atom. And then (keep again the blank lines), we write the displacement vectors as directly provided by Gaussian09. These displacement vectors have to also appear with increasing frequency. Let us recall that each row of each displacement vector corresponds to a vibrating atom. Thus, the order of the rows in each displacement vector has to be the same as that of the relaxed geometry. For example, the first row in our relaxed geometry is for uranium. Thus, the first row of all displacement vectors must also correspond to uranium. We suggest to introduce both the relaxed geometry and the displacement vectors in this input as directly provided by the Gaussian09 output. The code geom_dist.f90 provides the distorted geometries of each vibrational mode with the format required by the input simpre.dat in the package SIMPRE1.2 (ref. 12 in main text). Thus, the first row both in the relaxed geometry and in each displacement vector must always correspond to the metal. Moreover, all the metal-coordinating ligand atoms must be grouped together and must appear both in the relaxed geometry and in each displacement vector right after the first row (which corresponds to the metal). We suggest to build the Gaussian09 input such that the input geometry have the metal in the first row and the metal-coordinating ligand atoms grouped together in the following rows. Inside the code geom_dist.f90, there are some parameters: nmod is the number of vibrational modes, nato is the number of all vibrating atoms (including now also the hydrogen atoms whenever they are present), iesf decides whether the relaxed geometry and the distorted geometries are printed in Cartesian (Angstroms) or spherical coordinates (Angstroms, degrees, degrees), imax is the number of metal-coordinating ligand atoms plus one (i.e., this number exactly includes the metal and the metal-coordinating ligand atoms, only the rows from 2 to imax of the relaxed geometry and of the distorted geometries will be printed), rres is the radial displacement (Angstroms) to approach each metal-coordinating ligand atom to the metal ion (see REC model below), ceff is the effective charge to apply to each metal-coordinating ligand atom (see REC model below). The angles “the” and “phi” are used to rotate the relaxed geometry and the distorted geometries if desired. First, a clock-wise rotation of angle "the" (degrees) is performed around the Y=(0,1,0) axis. Then, an anticlock-wise rotation of angle "phi" (degrees) is performed around the Z=(0,0,1) axis. If this rotation is not desired, just set the=0.0d0 and phi=0.0d0. The rotation we use (the=84.9d0, phi=26.9d0 in degrees) is such that the two carbon rings that coordinate the metal become parallel to the XY plane. This rotation option only works when iesf=1. In the output geom_dist_output, one finds the relaxed geometry and the distorted geometries for each vibrational mode and for each value of the distortion coordinate. The rows containing the atom positions are ready to be used in the input simpre.dat in the package SIMPRE1.2 (ref. 12 in main text). To run geom_dist.f90, the library lapack is not required. We use the compiler gfortran and the command line: gfortran –o aa geom_dist.f90 (aa is the executable name).

All in all, for each mode j, several distorted geometries $\left\{\vec{v}_d^{\,j}\right\}_d$ are generated around the relaxed geometry $\vec{v}_{eq}$ by following the corresponding displacement vector $\vec{w}_j$. Then, we run a SIMPRE calculation at each distorted geometry $\vec{v}_j^{\,d}$ to determine the set of CFPs $A_k^q\left\langle r^k\right\rangle$ in cm^{-1}

by following the same procedure employed to determine $\left\{ \left(A_k^q \left\langle r^k \right\rangle \right)_{eq} \right\}_{k,q}$. Namely, to apply the same radial distance variations and charge magnitudes to the metal-coordinating atoms in these distorted geometries. Thus, for each mode j and each CFP we have a set of pairs $\left\{ \left(\left(A_k^q \left\langle r^k \right\rangle \right)_j^d , Q_j^d \right) \right\}_d$. In the folder CFPs-vs-Dist-Coord, one finds, for each vibrational mode, these CFPs (cm^{-1}) at each value of the distortion coordinate (Angstrom). The files in this folder are ready to plot each CFP evolution against the corresponding evolution of the distortion coordinate, for example, by using the Igor package. By fitting each plot "$\left(A_k^q \left\langle r^k \right\rangle \right)_j^d$ vs Q_j^d" to a second order polynomial and evaluating its second derivative at $Q_j = 0$ (let us recall that the second derivatives in Eq. 1 are evaluated at the relaxed geometry, which corresponds to $Q_j = 0$ for all j) we access $\left(\partial^2 A_k^q \left\langle r^k \right\rangle / \partial Q_j^2 \right)_{eq}$. The values of these second derivatives for each CFP and for each vibrational mode are provided in the input frrmcfpd2.inp (which can be found in the folder Home-Made-Codes). In general, to fit each plot "CFP vs Q_j", we use the smallest degree polynomial that provides the best visual and most reasonable fitting (see SI of ref. 9 in main text).

Transition rates

The system vibrations that perturb the equilibrium electronic structure (corresponding to the relaxed geometry) are considered as harmonic. To determine the probability per unit time (i.e., the transition rate) of driving a transition from a state of the system characterized by the ket $\left| E_i , n_j \right\rangle$, where $\left| E_i \right\rangle$ is a given initial eigenstate with energy E_i and the quantum number n_j describes the eigenstate of a given 1D harmonic vibrational mode j, to another state $\left| E_f , n_j \pm 1 \right\rangle$ either by emitting or by absorbing a phonon, where E_f is the energy of a given final eigenstate, is common to proceed by employing the so-called Fermi Golden Rule. This rule is usually prepared to incorporate a given expression of the phonon density of states. For example, the most employed phonon density of states is that of the Debye model, where this density is proportional to the square of the phonon frequency. Then, one integrates the transition rate over this phonon frequency up to the Debye cut-off, and the resultant expression depends on some parameters such as the crystal longitudinal and transverse sound velocities besides the Debye temperature. Let us recall that the current main goal is the development of fully ab-initio methodologies, in particular, by not assuming any specific form in the phonon density of states. This means being able to incorporate the vibrational spectrum as provided by a first-principles calculation. In solid state systems, vibrational energies are close enough so that it is considered they form an energy continuum. That is why the phonon frequency in the Debye model appears as a continuous variable -not discrete- which is subsequently integrated over a given real interval. On the contrary, a first-principles software will always provide a finite number of vibrations (each one with its harmonic frequency, reduced mass, force constant and displacement vector). These vibrations are the result of diagonalizing the so-called force matrix, which has always a finite size because computers only deal with finite quantities. To incorporate this finite set of vibrations into the transition rates, we need to replace the standard integral of

the phonon frequency over a real interval by a summation over this given set of vibrations. Thus, the transition rates we show below are the result of adapting the standard Fermi Golden Rule, where the integral over the phonon frequency have been substituted by a summation over all vibrational modes (ref. 3 in main text). Indeed, the spin-vibration coupling is calculated up to second order in perturbation theory. Thus, there no exist crossed interactions among different vibrational modes and the transition rate expressions are just a summation over independent vibrations (ref. 9 in main text). These transition rates have been derived under the so-called Born-Oppenheimer approximation, which assumes that the electronic and nuclear dynamics are uncoupled and it results in non-adiabatic electronic transitions.

Orbach transition rates:

This relaxation process is a finite sequence of direct transitions $|i\rangle \rightarrow |f\rangle$ where each one of them is driven by only one resonant phonon with the energy difference $|E_f - E_i|$. The process starts in an initial eigenstate with unity population. The spin is excited to higher intermediate eigenstates in the potential barrier through phonon absorption. Once the barrier has been crossed (either by overcoming the highest eigenstate or by tunneling), this is followed by a cascade of de-excitations until reaching a final eigenstate through phonon emission.

Phonon absorption: $$\gamma_{fi} = \frac{2\pi}{\hbar}\sum_{j=1}^{R}\left[\left|\langle i|\hat{H}_j|f\rangle\right|^2 \left|\langle n_j - 1|\varepsilon_j|n_j\rangle\right|^2 \rho_j\left(|E_i - E_f|\right)\right] \quad \text{Eq. S1}$$

Phonon emission: $$\gamma_{if} = \frac{2\pi}{\hbar}\sum_{j=1}^{R}\left[\left|\langle i|\hat{H}_j|f\rangle\right|^2 \left|\langle n_j + 1|\varepsilon_j|n_j\rangle\right|^2 \rho_j\left(|E_i - E_f|\right)\right] \quad \text{Eq. S2}$$

R is the number of vibrational modes.

Second-order Raman transition rates:

The transition from $|i\rangle$ to $|f\rangle$ is not direct but driven through an intermediate eigenstate $|c\rangle$, and involves two resonant phonons with the energy differences $|E_c - E_i|$ and $|E_f - E_c|$. The first phonon j mixes $|i\rangle$ with $|c\rangle$, while the second one l mixes $|c\rangle$ with $|f\rangle$. Now, the case $E_i = E_f$ will have a certain transition rate whose value is not necessarily zero. Given $|i\rangle$ and $|f\rangle$, we include in the transition rate expression all intermediate eigenstates $|c\rangle$ but the ones with an energy E_c equal to either E_i or E_f (ref. 3 in main text). Thus, given $|i\rangle$ and $|f\rangle$, for each $|c\rangle$ only one of the following four options regarding the order in the energies is possible: (i) $E_i < E_c > E_f$, (ii) $E_i > E_c < E_f$, (iii) $E_i > E_c > E_f$, (iv) $E_i < E_c < E_f$. The transition rate expression is as follows:

$$\gamma = \frac{2\pi}{\hbar}\sum_{\substack{c=1\\ E_c \neq E_i, E_f}}^{2J+1}\sum_{j=1}^{R}\sum_{l=1}^{R}\left[\frac{\left|\langle c|\hat{H}_j|i\rangle\right|^2 \left|\langle f|\hat{H}_l|c\rangle\right|^2}{|E_c - E_i||E_f - E_c|}\Theta(c,j)\Theta(c,l)\rho_j\left(|E_c - E_i|\right)\rho_l\left(|E_f - E_c|\right)\right]$$

Eq. S3

In case of (i), the phonon j is absorbed and the phonon l is emitted; thus $\Theta(c,j)=\left|\langle n_j-1|\varepsilon_j|n_j\rangle\right|^2$ and $\Theta(c,l)=\left|\langle n_l+1|\varepsilon_l|n_l\rangle\right|^2$. In case of (ii), the phonon j is emitted and the phonon l is absorbed; thus $\Theta(c,j)=\left|\langle n_j+1|\varepsilon_j|n_j\rangle\right|^2$ and $\Theta(c,l)=\left|\langle n_l-1|\varepsilon_l|n_l\rangle\right|^2$. In case of (iii), the phonon j is emitted and the phonon l is emitted; thus $\Theta(c,j)=\left|\langle n_j+1|\varepsilon_j|n_j\rangle\right|^2$ and $\Theta(c,l)=\left|\langle n_l+1|\varepsilon_l|n_l\rangle\right|^2$. In case of (iv), the phonon j is absorbed and the phonon l is absorbed; thus $\Theta(c,j)=\left|\langle n_j-1|\varepsilon_j|n_j\rangle\right|^2$ and $\Theta(c,l)=\left|\langle n_l-1|\varepsilon_l|n_l\rangle\right|^2$.

<u>Matrix elements of the strain tensor</u>:

$$\left|\langle n_j-1|\varepsilon_j|n_j\rangle\right|^2=\frac{1}{e^{\hbar\omega_j/k_BT}-1}\qquad \left|\langle n_j+1|\varepsilon_j|n_j\rangle\right|^2=\frac{1}{1-e^{-\hbar\omega_j/k_BT}}\quad \text{Eq. S4}$$

These matrix elements describe the strain suffered by the lattice, which is encoded in the so-called strain tensor ε_j, when the vibrational mode j absorbs or emits a phonon, respectively. The vibration bath is considered to be thermalized, i.e., its dynamics is much faster than that of the magnetic relaxation. Thus, these matrix elements are proportional to the Bose-Einstein statistics of the given vibrational mode j, and depend only on temperature (ref. 22 and 24 in main text).[2] Note that when temperature $T\to 0$ the left matrix element in Eq. S4 vanishes, but not the right term which tends to 1. This means that some transition rates do not necessarily vanish as $T\to 0$, and thus the spin is expected to relax even at very low temperature.

<u>Distribution of phonon energies</u>:

$$\rho_j(\hbar\omega)=\frac{1}{\sigma\sqrt{2\pi}}\exp\left(-\frac{1}{2}\left(\frac{\hbar\omega-\hbar\omega_j}{\sigma}\right)^2\right),\ j=1,\ldots,R\quad \text{Eq. S5}$$

There is another modification implemented in the above transition rates. The original expressions contain the Dirac delta function $\delta(\hbar\omega-\hbar\omega_j)$, where $\hbar\omega=|E_f-E_i|$ is the energy difference between the final and the initial eigenstates. The conservation of energy implies that $\hbar\omega$ must equal the phonon energy $\hbar\omega_j$ of a given vibrational mode j. Otherwise, both $\delta(\hbar\omega-\hbar\omega_j)$ and the corresponding transition rate vanish, i.e., there is no spin transition. As said above, first-principles packages provide a discrete vibrational spectrum. Thus, it is quite unlikely to find a vibrational mode whose phonon energy exactly matches a given energy difference $|E_f-E_i|$, and hence one would not observe any spin relaxation. To solve this issue (ref. 3 in main text), the Dirac delta function is replaced by a Gaussian convoluted spectrum

around the phonon energy $\hbar\omega_j$ of the given vibrational mode j. In other words, we let the phonon energy have an uncertainty width around its value $\hbar\omega_j$. This width is determined by the standard deviation parameter σ, and can be estimated by inspecting the experimental IR and Raman vibrational spectra (the full-width-half-maximum linewidth is twice as much as σ) (ref. 3 in main text). This parameter has to be estimated carefully, since a too small value makes the Gaussian convoluted spectrum become too much similar to a delta function, and no relaxation is observed. On the contrary, a too large value means a continuously flat vibrational spectrum, which is not observed for molecular systems. Our case study just aims to demonstrate the methodology that we are proposing, and we have no experimental vibrational spectra of $[U(Cp^{ttt})_2]^+$. Thus, we decided to employ the same value as in $[Dy(Cp^{ttt})_2]^+$, which is $\sigma \sim 10 cm^{-1}$.

Resolution of the master equation

It is important to stress that the master equation in Eq. 2 is not invariant by time reversal. Thus, it is only valid for large enough times when irreversibility in the macroscopic system has been established. Irreversibility is reached above the time scale in which the relevant system-environment collisions occur. Since spin-vibration interactions are much faster than relaxation in magnetic molecules, we can safely assume the attainment of this macroscopic irreversibility (ref. 22 in main text). A more complete description of relaxation would need to use density matrix formalism, since the employed picture consists in a spin population flowing among the several eigenstates, which thus disregards any coherent superposition of them.

Orbach process:

We explain now how to solve the master equation in Eq. 2 (ref. 3, 22, 23 in main text). For that, we make use of the Orbach transition rates in Eq. S1 and Eq. S2. First, we need to build the so-called master matrix Γ from the transition rates, whose size is $(2J+1)\times(2J+1)$ (J is the ground electron spin quantum number of the magnetic metal ion). As explained in the main text, after diagonalizing the equilibrium crystal field Hamiltonian we obtain the lowest $2J+1$ eigenstates, which are truncated to the $|m_J\rangle$ components of the ground J multiplet and subsequently renormalized in the case of studying a U^{3+}-based molecular magnet. Then, these eigenstates $|e\rangle$ must be ordered. In our case, the order we consider is the one provided in the input rates.inp for the code rates.f (see section "Fortran code to evaluate magnetic relaxation dynamics"). In this input, we write the eigenstates row-wise; the row 1 is for the first eigenstate $|e_1\rangle$ and the row $2J+1$ is for the last one $|e_{2J+1}\rangle$. The master matrix Γ is:

Γ	$\|e_1\rangle$	$\|e_2\rangle$	...	$\|e_{2J}\rangle$	$\|e_{2J+1}\rangle$
$\|e_1\rangle$					
$\|e_2\rangle$					
...					
$\|e_{2J}\rangle$					
$\|e_{2J+1}\rangle$					

First, we fill in the off-diagonal elements. Given initial and final eigenstates $|i\rangle$ and $|f\rangle$, the positions of $|i\rangle$ and $|f\rangle$ in Γ determine the column and the row of the off-diagonal element to fill in, resp. If $E_f > E_i$, we use the transition rate γ_{fi} (phonon absorption). If $E_f < E_i$, we use the transition rate γ_{if} (phonon emission). If $E_f = E_i$, we use $\gamma = 0$ (ref. 3 in main text, we are not including quantum tunneling of magnetization due to the action of a spin bath). Now, if the off-diagonal element ($|f\rangle, |i\rangle$) has been filled with γ_{fi}, the symmetric off-diagonal element ($|i\rangle, |f\rangle$) is filled with γ_{if}. If the off-diagonal element ($|f\rangle, |i\rangle$) has been filled with γ_{if}, the symmetric off-diagonal element ($|i\rangle, |f\rangle$) is filled with γ_{fi}. If the off-diagonal element ($|f\rangle$, $|i\rangle$) has been filled with $\gamma = 0$, the symmetric off-diagonal element ($|i\rangle, |f\rangle$) is also filled with $\gamma = 0$. Each diagonal element in a given position $\left(|e\rangle, |e\rangle\right)$ is the negative summation of the off-diagonal elements in the given $|e\rangle$ column. By numerical diagonalization, $2J+1$ real eigenvectors $\left(\varphi_{e_1,k}, \ldots, \varphi_{e_{2J+1},k}\right)$ and eigenvalues $-1/t_k$ are obtained, where t_k are the positive $2J+1$ relaxation times of the system ($-1/t_k$ are thus the $2J+1$ relaxation rates of the system). One of these rates $-1/t_{k_0}$ is always zero, and corresponds to the situation in which the system has reached the thermal equilibrium. From these formalism, an expression for the total magnetization $M(t)$ as a function of time is obtained, Eq. S6, which depends on the population $0 \le p_e(t) \le 1$ and on the magnetization M_e of each eigenstate $|e\rangle$.

The expression of $p_e(t)$ is shown in Eq. S7, and is a finite sum proportional to the $2J+1$ exponential functions e^{-t/t_k}. The coefficients $\left\{\lambda_k\right\}_{k=1}^{2J+1}$ are obtained by solving the linear equation system in Eq. S7 after setting $t=0$ and introducing the $2J+1$ initial populations $0 \le p_e(t=0) \le 1$. The magnetizations M_e are calculated as the derivatives $M_e = -\frac{\partial}{\partial B}\langle e|\hat{H}_{ZE}|e\rangle$ evaluated at $B=0$, where B is a static magnetic field and $\hat{H}_{ZE}$ is the Zeeman Hamiltonian.[3] After applying the Hellmann-Feynman theorem, M_e can be rewritten as $M_e = -\langle e|\frac{\partial \hat{H}_{ZE}}{\partial B}|e\rangle$. We consider that the given magnetic molecule shows an axial anisotropy, with an axis either easy or hard that defines the Z axis. The magnetic field is applied in this Z direction, and thus the Zeeman Hamiltonian is $\hat{H}_{ZE} = \mu_B g B \hat{J}_z$, where μ_B is the Bohr magneton and g is the free-ion Landé factor. Hence, $M_e = -\mu_B g \langle e|\hat{J}_z|e\rangle$. Let us write the eigenstate $|e\rangle$ in the basis set of the ground J multiplet as $|e\rangle = \sum_{j=1}^{2J+1} c_e(j)|j-J-1\rangle$, where $c_e(j)$ are complex coefficients such that $|e\rangle$ is normalized. After some algebra, it is easy to obtain the expression $\langle e|\hat{J}_z|e\rangle = \sum_{j=1}^{2J+1} |c_e(j)|^2 (j-J-1)$ for the expectation value of the z component of the electron spin operator $\hat{J}$.

$$M(t)=\sum_{e=1}^{2J+1} p_e(t) M_e \quad \text{Eq. S6}$$

$$p_e(t)=\sum_{k=1}^{2J+1} \lambda_k \varphi_{e,k} e^{-t/t_k} \quad \text{Eq. S7}$$

In practice, $2J-1$ of the exponential functions in Eq. S7 vanish quickly (there are $2J-1$ relaxation rates that are extremely fast), and $M(t)$ behaves as a single decaying exponential (corresponding to the only one slow relaxation rate) plus a constant derived from $-1/t_{k_0}=0$. This fact is due to the double-well anisotropy. Indeed, each one of these $2J-1$ fast relaxation rates describes spin dynamics inside a given side of the potential barrier. On the contrary, the spin jump over the barrier takes a much longer time and is accounted for by the slowest relaxation rate.

Since g is independent of the script e, we redefine the magnetization M_e as $\tilde{M}_e = M_e/\mu_B g$. Thus, $\tilde{M}_e = -\sum_{j=1}^{2J+1} \left|c_e(j)\right|^2 (j-J-1)$. We can also redefine the total magnetization $M(t)$ as $\tilde{M}(t)=M(t)/\mu_B g$. Thus, $\tilde{M}(t)=-\sum_{e=1}^{2J+1}\left(p_e(t)\sum_{j=1}^{2J+1}\left|c_e(j)\right|^2 (j-J-1)\right)$. By fitting the plot "$\tilde{M}(t)$ vs t" to an exponential function $f(t)=a+be^{-t/\tau}$, the overall Orbach-mediated magnetic relaxation time τ is extracted, which is dominated by the slowest relaxation rate $-1/t_k$ at the working temperature as explained.

<u>Second-order Raman process</u>:

Each off-diagonal element (column $|i\rangle$, row $|f\rangle$) of the master matrix is filled by calculating the transition rate given in Eq. S3. The diagonal elements are also built by adding the elements of the corresponding column and changing the sign of the summation. The master matrix is diagonalized, which allows us reaching the total magnetization whose time decay is fitted to a single exponential function to extract the Raman-mediated magnetic relaxation time at the working temperature.

In the following table, we show the second-order Raman relaxation time calculated at different temperatures for $[U(Cp^{ttt})_2]^+$.

Table S1. Thermal evolution of the second-order Raman relaxation time for $[U(Cp^{ttt})_2]^+$.

Temperature (K)	Second-order Raman Relaxation Time (s)
11	$1{,}3\cdot10^{11}$
12	$2{,}0\cdot10^{10}$
13	$4{,}1\cdot10^{9}$
14	$1{,}0\cdot10^{9}$
15	$3{,}2\cdot10^{8}$
20	$5{,}2\cdot10^{6}$

25	$4,4\cdot10^5$
30	$8,4\cdot10^4$
40	$1,0\cdot10^4$
50	$2,7\cdot10^3$

The reason to calculate the Orbach and second-order Raman relaxation times up to 50 K is because at this temperature the Orbach relaxation time (Fig. 3) is already reaching the lowest value for a relaxation time that is experimentally observed, which is around 10^{-5}-10^{-6} s. The reason to calculate the relaxation times above 6 K in case of the Orbach process (Fig. 3) and above 11 K in case of the second-order Raman process is because below these temperatures the smallest relaxation rates (eigenvalues of the corresponding master matrix) cannot be distinguished from the computational numerical noise. For example, below these temperatures one finds that the two smallest relaxation rates are both positive. It is important to note that only one relaxation rate $-1/t_{k_0}$ may be positive as much, and this rate in fact should be strictly zero (see above). Nevertheless, because of the computational numerical noise, it may have a rather non-zero value. That is why we introduce the thresholds eot (in case of the Orbach master matrix) and ert (in case of the second-order Raman master matrix), which have to be given a value such that they are above the magnitude of the smallest relaxation rate and below the magnitude of the second smallest relaxation rate (see section "Fortran code to evaluate magnetic relaxation dynamics"). The remaining $2J$ relaxation rates should be all of them always real and negative. Below these temperatures, we have also found (i) imaginary relaxation rates (although they are rather small and comparable to the computational numerical noise), (ii) the two smallest relaxation rates which are too similar to be distinguished and to decide which one should be strictly zero, and (iii) the smallest non-zero relaxation rates too small that makes the magnetization not to decay with time (which is interpreted as an infinite relaxation time).

Determination of relaxation pathways and identification of vibrations promoting relaxation

To get further insight into the spin dynamics, a proper re-cast of the Orbach master matrix Γ provides the different relaxation pathways at the working temperature. In this master matrix, the ket that determines a given column is the initial eigenstate $|i\rangle$ of a given direct transition, while the ket of a given row is the final eigenstate $|f\rangle$ of the same direct transition. At time $t=0$, we place all the initial population in the $[U(Cp^{ttt})_2]^+$ eigenstate with $\langle \hat{J}_z \rangle = -4.15$, see Fig. 4. Note that in Fig. 4 the eigenstates are not ordered from left to right by increasing $\langle \hat{J}_z \rangle$ value. Nevertheless, let us consider the appearance order from left to right of each one of these eigenstates in this figure. Thus, the eigenstate with $\langle \hat{J}_z \rangle = -4.15$ is first, the one with $\langle \hat{J}_z \rangle = -1.28$ is second, and so on until the final eigenstate with $\langle \hat{J}_z \rangle = +4.15$. We only consider those relaxation pathways whose direct transitions are always to a final eigenstate $|f\rangle$ which is to the right of the given initial eigenstate $|i\rangle$ in Fig. 4 (ref. 3 in main text). For example, from the eigenstate with $\langle \hat{J}_z \rangle = -4.15$ the direct transitions can be to any other

eigenstate but the one with $\langle \hat{J}_z \rangle = +4.15$ (let us recall that in this approach we are not considering direct transitions between degenerate eigenstates), while from the eigenstate with $\langle \hat{J}_z \rangle = +0.64$ the direct transitions can only be to the one with $\langle \hat{J}_z \rangle = +1.28$ or to the one with $\langle \hat{J}_z \rangle = +4.15$. Sometimes (ref. 3 in main text), these equilibrium eigenstates may be pure in the sense that their $\hat{J}_z$ expectation values coincide with the several $m_J = -J,...,+J$ values. In this case, they would be ordered from left to right by increasing $\langle \hat{J}_z \rangle$ value, and to re-cast the Orbach master matrix only those direct transitions that increase the $\langle \hat{J}_z \rangle$ value (but the ones between degenerate eigenstates) would be considered, where the $\langle \hat{J}_z \rangle = -J$ eigenstate would be the one with unit population at $t = 0$. In our case, to do the re-cast, in the Orbach master matrix we only need to keep those entries that correspond to a direct transition between non-degenerate eigenstates, where the final eigenstate $|f\rangle$ is found to the right of the initial eigenstate $|i\rangle$ in Fig. 4. The remaining entries, including the ones in the main diagonal, are now filled with a zero (transitions from a given eigenstate to the same eigenstate are excluded). In the input rates.inp of the code rates.f, right after writing the initial populations at time $t = 0$, one writes a table whose size is the same as that of the Orbach master matrix (see section "Fortran code to evaluate magnetic relaxation dynamics"). We write "1" at those positions that correspond to those direct transitions that are kept; at the remaining positions we write "0". The re-casted Orbach master matrix appears in the output rates.out, in the section "Orbach relaxation pathway". Once the Orbach master matrix is re-casted, it needs now to be normalized in terms of percentages. The normalized re-casted Orbach master matrix appears in rates.out right after the re-casted Orbach master matrix. For that, right after the re-casting table just mentioned, we first write in rates.inp the total number of possible direct transitions between consecutive eigenstates from left to right in Fig. 4. Since there are 10 eigenstates, this total number is 9. Then, after this number we write the order of the equilibrium eigenstates as they appear in Fig.4 from left to right. The rightmost eigenstate $\langle \hat{J}_z \rangle = +4.15$ is excluded because there are no more eigenstates to the right, hence, there are no possible direct transitions from this eigenstate to keep. This order depends on the order with which the eigenstates were introduced at the beginning of rates.inp. In this input, we wrote the eigenstates in this order: 1 $\langle \hat{J}_z \rangle = -4.15$, 2 $\langle \hat{J}_z \rangle = +4.15$, 3 $\langle \hat{J}_z \rangle = -1.28$, 4 $\langle \hat{J}_z \rangle = +1.28$, 5 $\langle \hat{J}_z \rangle = -0.64$, 6 $\langle \hat{J}_z \rangle = +0.64$, 7 $\langle \hat{J}_z \rangle = -1.49$, 8 $\langle \hat{J}_z \rangle = +1.49$, 9 $\langle \hat{J}_z \rangle = -1.78$, 10 $\langle \hat{J}_z \rangle = +1.78$. The order we need to write in rates.inp is: $\langle \hat{J}_z \rangle = -4.15$ -> $\langle \hat{J}_z \rangle = -1.28$ -> $\langle \hat{J}_z \rangle = -0.64$ -> $\langle \hat{J}_z \rangle = -1.49$ -> $\langle \hat{J}_z \rangle = -1.78$ -> $\langle \hat{J}_z \rangle = +1.78$ -> $\langle \hat{J}_z \rangle = +1.49$ -> $\langle \hat{J}_z \rangle = +0.64$ -> $\langle \hat{J}_z \rangle = +1.28$ -> $\langle \hat{J}_z \rangle = +4.15$. Thus, we write in rates.inp the sequence 1 3 5 7 9 10 8 6 4 (note that the number 2 which corresponds to the $\langle \hat{J}_z \rangle = +4.15$ eigenstate is not present). The normalization in terms of percentages is based on the order of the sequence 1 3 5 7 9 10 8 6 4, and is as follows. These numbers in this sequence have now to be read as if they are referring to the corresponding columns of the re-casted Orbach master matrix (let us recall that the

eigenstates that determine the several columns of this matrix are the initial eigenstates in the direct transitions). The first position in this sequence corresponds to the eigenstate with unity population at time $t = 0$, and the populations of all direct transitions that departure from this eigenstate must amount to 100%. Thus, we sum all the elements in the column 1 of the re-casted Orbach master matrix, divide each one of these elements by this sum, and multiply each result by 100. After this process, each element of the first column in the normalized re-casted Orbach master matrix gives the population that flows from the eigenstate 1 ($\langle \hat{J}_z \rangle = -4.15$) to the rest of the eigenstates. In particular, the element (3,1) (row 3 and column 1) gives the population $P_{1\to3}$ that flows from the eigenstate 1 ($\langle \hat{J}_z \rangle = -4.15$) to the eigenstate 3 ($\langle \hat{J}_z \rangle = -1.28$). Now, the process is repeated in the column 3 of the re-casted Orbach master matrix. We add all the elements in the column 3, divide each element by this sum, and now multiply each result by $P_{1\to3}$. In this way, the sum of all the incoming populations to the eigenstate 3 ($\langle \hat{J}_z \rangle = -1.28$) equals the sum of all the outcoming populations from the same eigenstate, which is not necessarily 100. Now, each element of the third column in the normalized re-casted Orbach master matrix gives the population that flows from the eigenstate 3 ($\langle \hat{J}_z \rangle = -1.28$) to the rest of the eigenstates that are to the right in Fig. 4 (but the one with $\langle \hat{J}_z \rangle = +1.28$). The process is repeated again now with the column 5 of the re-casted Orbach master matrix, and finishes with the column 4 of this matrix. It is possible that the sum of the elements of a given column of the re-casted Orbach master matrix be below the computational numerical precision, which means this sum is as if it was zero. If this sum is zero from the point of view of the employed computer, each element of the given column can also be considered to be zero, since they are all always positive. Thus, it is not possible to divide these elements by the just mentioned sum. We introduce the threshold pot = 1.0d-16 in the code rates.f to decide when this sum has to be considered as zero. In this case, all the elements of the given column in the re-casted Orbach master matrix are set to be zero.

Once the re-casted Orbach master matrix has been normalized, each direct transition that compose each relaxation pathway can now be decomposed into the contributions from the several vibrational modes. Indeed, let us recall that each direct transition corresponds to an element of the re-casted Orbach master matrix. These elements are in fact transition rates, which are a sum over the several vibrational mode contributions, see Eq. S1 and Eq. S2. What we do in rates.f is to normalize each vibrational mode contribution in terms of percentages, i.e., we divide each contribution by the value of the given transition rate and then multiply the result by 100. If this percentage is above a threshold (between 0.0 and 100.0), then the vibrational mode number along with its contribution percentage is printed in the output rates.out (see section "Fortran code to evaluate magnetic relaxation dynamics"). This threshold is the last real number written in the input rates.inp. If a given transition rate in the re-casted Orbach master matrix is below the computational numerical precision, it can be considered to be zero. This means that each vibrational mode contribution to this transition rate can also be considered to be zero, since all of the contributions are positive. In this case, no vibrational modes corresponding to the given transition rate are shown in rates.out. To decide when a given transition rate has to be considered as zero, we use the threshold mct=1.0d-16 in the code rates.f. Once the most contributing vibrational modes are identified, we can now visually inspect how the molecule vibrates to check which atomic movements are involved. Then, chemical modifications in the molecular structure can be rationally proposed in order to remove these

modes and suppress magnetic relaxation as much as possible, with the hope of improving the molecular magnet performance. In the outputs that are placed in the folder Orbach-rp-Raman, one can find those vibrational modes that contribute (>10%) to each direct transition of the Orbach-mediated relaxation pathways (see Fig. 4) at the several working temperatures.

Above T = 30 K, the thermally activated relaxation is at play. The spin population flows through excited doublets by absorption and emission of phonons (Fig. 4). We identify up to six vibrations involved in this relaxation mechanism. These are the calculated vibrations 16, 17, 18, 19, 20 ,21, with harmonic frequencies ν_{16} = 135.0115 cm^{-1}, ν_{17} = 136.8658 cm^{-1}, ν_{18} = 170.0364 cm^{-1}, ν_{19} = 172.5580 cm^{-1}, ν_{20} = 175.4401 cm^{-1}, ν_{21} = 175.7696 cm^{-1} (see folder Animations), which closely match the gaps between the equilibrium ground and first excited doublets (159.3 cm^{-1}), and first and second excited doublets (171.7 cm^{-1}). The vibration 16 is a rocking-like deformation of the two Cp^{ttt} rings: the two hydrogen atoms bounded to each Cp^{ttt} ring moves towards and away from the U^{3+} ion. As a side effect, there are also rigid movements of the terc-butyl substituents. This kind of vibration was also identified in a previous study on the molecular magnet $[Dy(Cp^{ttt})_2]^+$ (calculated 64-67 vibrations of ref. 3 in main text) as the one promoting the first step in the most likely relaxation pathway from the ground doublet to the first excited doublet. It was proposed to substitute these two hydrogen atoms in the Cp^{ttt} rings by bulkier substituents in order to block this vibration and, in fact, this substitution was carried out in a subsequent study (ref. 5 in main text). This modification worked since this vibration is no longer observed and, indeed, the blocking temperature is increased from 60 K to 80 K. The experimental effective barrier U_{eff} is also increased from 1223 cm^{-1} to 1541 cm^{-1}. The vibration 17 involves kind of rigid movements in the terc-butyl substituents. The modes 18 and 21 are symmetric and antisymmetric breathing vibrations: the two Cp^{ttt} rings moves towards and moves away from the U^{3+} ion at once and out of phase, respectively, and are also found in the recently reported molecular magent Dy-5* (vibrations 66, 67, 68 of ref. 5 in main text). This vibration could be suppressed by bounding these two Cp^{ttt} rings, such as it happens in stapled bis-phthalocyanines. The vibrations 19 and 20 involve methyl rotations in the terc-butyl substituents. These rotations could be partially suppressed if one replaces the methyl groups $–CH_3$ by the heavier fluorinated analogs $–CF_3$.

Fortran code to evaluate magnetic relaxation dynamics

In the folder Home-Made-Codes one finds the code rates.f, which is aimed to evaluate magnetic relaxation dynamics as we explain below, and the three inputs it needs, which are already prepared such as we used them: rates.inp, matrix.inp, frrmcfpd2.inp (matrix.inp is found in the subfolder Pert-Ham-Temp for each temperature, do not forget to rename this input before running rates.f). The code can generate up to three outputs: rates.orbach.out (see the folder Orbach-mag), rates.raman.out, and rates.out (see the folder Orbach-rp-Raman). To run rates.f, the library lapack is required. To compile, we use the compiler gfortran and the command line is: gfortran –o aa –llapack rates.f, where aa is the executable name. A useful information to run the codes is the numerical value of the ground electron spin quantum number J of the selected magnetic metal ion: $J\left(Ce(III)\right)=5/2$, $J\left(\mathrm{Pr}(III)\right)=4$, $J(Nd(III),U(III))=9/2$, $J\left(Pm(III)\right)=4$, $J\left(Sm(III)\right)=5/2$, $J\left(Tb(III)\right)=6$, $J\left(Dy(III)\right)=15/2$, $J\left(Ho(III)\right)=8$, $J\left(Er(III)\right)=15/2$, $J\left(Tm(III)\right)=6$, $J\left(Yb(III)\right)=7/2$. We describe now these inputs, the code and the outputs:

frrmcfpd2.inp: this input contains the harmonic frequencies with increasing energy (cm^{-1}, as directly provided by the Gaussian09 output uranocenium-r1.log), reduced masses accordingly ordered with the harmonic frequencies (chemical atomic mass units, as directly provided by the Gaussian09 output uranocenium-r1.log), the second derivatives of the CFPs $A_k^q \left\langle r^k \right\rangle$ respect to the distortion coordinate of each vibrational mode evaluated at the relaxed geometry (cm^{-1}·Å^{-2}, the order of these derivatives is (2,0), (2,1), (2,-1), (2,2), (2,-2), (4,0), (4,1), (4,-1), (4,2), (4,-2), (4,3), (4,-3), (4,4), (4,-4), (6,0), (6,1), (6,-1), (6,2), (6,-2), (6,3), (6,-3), (6,4), (6,-4), (6,5), (6,-5), (6,6), (6,-6), where the parenthesis (k,q) represents the scripts k = 2, 4, 6, q = -k,…, +k), and the CFPs $\left(A_k^q \left\langle r^k \right\rangle\right)_{eq}$ determined at the relaxed geometry (cm^{-1}, given in the same order as that in each one of the second derivatives). Actually, rates.f only needs the harmonic frequencies from this input.

matrix.inp: this input contains the perturbing Hamiltonian $\hat{H}_j = \sum_{k=2,4,6} \sum_{q=-k}^{k} \Delta\left(A_k^q \left\langle r^k \right\rangle\right)_j (T) \eta_k \hat{O}_k^q$ of each vibrational mode j at a given temperature T (see Eq. 1 in main text), where $\eta_2 = \alpha$, $\eta_4 = \beta$, $\eta_6 = \gamma$ are the Stevens factors and $\hat{O}_k^q$ are the Stevens equivalent operators. These Hamiltonians in matrix.inp follow the harmonic frequencies order in frrmcfpd2.inp. Each perturbing Hamiltonian is a $(2J+1)\times(2J+1)$ complex matrix with the same units as the parameters $\Delta\left(A_k^q \left\langle r^k \right\rangle\right)_j (T)$ (here, in cm^{-1}), written in the ordered basis set $\left\{\left|-J\right\rangle,\ldots,\left|+J\right\rangle\right\}$, where J is the ground electron spin quantum number. The parameters $\Delta\left(A_k^q \left\langle r^k \right\rangle\right)_j (T)$ are determined by running the cfppert.f code (see the section "Fortran codes to calculate CFPs thermal evolution").

rates.inp:

The first row contains the lowest $2J+1$ energies (cm^{-1}) calculated at the relaxed geometry, i. e., those obtained by diagonalizing the equilibrium crystal field Hamiltonian $\hat{H}_{eq} = \sum_{k=2,4,6} \sum_{q=-k}^{k} \left(A_k^q \left\langle r^k \right\rangle\right)_{eq} \eta_k \hat{O}_k^q$, which is built with the CFPs $\left(A_k^q \left\langle r^k \right\rangle\right)_{eq}$ of the relaxed geometry. Let us recall that for U^{+3}-based molecular magnets the diagonalization is performed by using the CONDON package (ref. 20 in main text), where the Hamiltonian includes both the ground and excited J multiplets and the CFPs must be introduced in Wybourne notation since this software uses a rather different implementation of the crystal field operators.

The following $2J+1$ rows contain the lowest $2J+1$ eigenstates calculated at the relaxed geometry, i. e., those obtained by diagonalizing the equilibrium crystal field Hamiltonian $\hat{H}_{eq}$ (see above). The coefficients are complex and such that each eigenstate is normalized. These $2J+1$ rows are accordingly ordered with the corresponding $2J+1$ energies. Let us recall that for U^{3+}-based molecular magnets (see above) the lowest $2J+1$ eigenstates obtained by the CONDON package must be truncated to the $\left|m_J\right\rangle$ components of the ground J multiplet and then renormalized. Each eigenstate –either truncated and then renormalized or not- is written from left to right in the ordered basis set given by $\left|-J\right\rangle$ (leftmost coefficient),…, $\left|+J\right\rangle$ (rightmost coefficient), where J is the ground electron spin quantum number.

The following row contains the initial population, at time $t=0$, of the lowest $2J+1$ eigenstates. These initial populations are accordingly ordered with the corresponding $2J+1$ eigenstates. Each initial population must be a number between 0.0 and 1.0. The summation of all initial populations must be equal to 1. In our case study, the initial population is all in the truncated eigenstate with $\langle J_z \rangle = -3.99$. For the remaining rows, see the section "Determination of relaxation pathways and identification of vibrations promoting relaxation".

rates.f:

The variables iorb, icpr, iram are switches to turn on or to turn off the calculation of the time evolution of magnetization by using the Orbach transition rates (output: rates.orbach.out), the calculation of the relaxation pathways and determination of the involved vibrations (output: rates.out), and the calculation of the time evolution of magnetization by using the second-order Raman transition rates (output: rates.raman.out), respectively. If one wants to set icpr = 1, then it is mandatory to set iorb = 1.

The variable temp is the working temperature (K), while sigma (cm^{-1}) is the Gaussian width employed in the transition rates (see section "Transition rates"). The number of time points to calculate the magnetization and to show both in rates.orbach.out and in rates.raman.out is npmag. The variables tim (s) and tfm (s) are the initial and final time points, respectively. Depending on which is the time scale where magnetization decays, one will have to change tim and tfm until detecting the magnetization decay with time. The number of vibrational modes is nmodos, dj is the numerical value of the ground electron spin quantum number J, and idtot is $2J+1$.

The following variables are thresholds which are necessary to take some important decisions. As it is known (see section "Resolution of the master equation"), one of the eigenvalues of the master matrix is strictly zero. Because of computational numerical noise and since computers work with finite precision arithmetic, the smallest eigenvalue is not zero (see description of the output rates.out below). The numerical value of this eigenvalue may be taken as a measure of the computational numerical noise. Since this smallest eigenvalue must be zero, we give the thresholds eot (s^{-1}) and ert (s^{-1}) a value a bit above the absolute value of this smallest eigenvalue. The threshold eot works when using the Orbach transition rates, while ert works in case of using the second-order Raman transition rates. One can first run a rates.f calculation, check the eigenvalues and then decide a value for both eot and ert. The threshold edt (cm^{-1}) is used to decide whether the energies of two given eigenstates are different. For the description of pot and mct see the section "Determination of relaxation pathways and identification of vibrations promoting relatxation".

rates.out: this output is broken down into three sections: "Orbach mechanism", "Orbach relaxation pathway", and "Raman mechanism". These sections will appear or not depending on which switches iorb, icpr, iram are turned on. At the beginning of each section, one can read the working temperature.

In the sections "Orbach mechanism" and "Raman mechanism" one finds first the eigenvalues of the master matrix. These eigenvalues must be always real and negative (but maybe the smallest one which, as said above, is technically zero). They are printed as complex numbers to check whether the imaginary part is zero. Then, the eigenvectors of the master matrix are printed row-wise and appear in the same order as that of the corresponding eigenvalues. Let us recall that the master matrix is built in the ordered basis set given by the lowest $2J+1$ eigenstates

calculated at the relaxed geometry as provided in the input rates.inp (see section "Resolution of the master equation"). Thus, the components of each eigenvector follow the same order as that of these lowest $2J+1$ eigenstates in the rates.inp input. Then, one finds the coefficients $\{\lambda_k\}_{k=1}^{2J+1}$, which are the solution of a linear equation system built from the initial populations (see section "Resolution of the master equation"). Each one of these coefficients appear in Eq. S6 along with its corresponding eigenvalue $-1/t_k$. In the rates.out output, these coefficients are shown in the same order as that of the corresponding eigenvalues. Then, the lowest $2J+1$ energies calculated at the relaxed geometry (provided in the rates.inp input) are printed, along with the J_z expectation values of the corresponding eigenstates provided also in the rates.inp input. To end up these two sections, one finds the master matrix.

In the section "Orbach relaxation pathway" one first finds the re-casted Orbach master matrix, which is subsequently normalized in terms of percentage (see section "Determination of relaxation pathways and identification of vibrations promoting relaxation"). To end up, it is shown those vibrational modes that contribute to the Orbach transition rates in the re-casted Orbach master matrix above a given threshold. This threshold is provided in the rates.inp input (see section "Determination of relaxation pathways and identification of vibrations promoting relaxation"). First, each pair i -> f identifies the relevant Orbach transition rate which is the component of the re-casted Orbach master matrix in the column "i" and in the row "f" (see section "Resolution of the master equation"). After i -> f, those modes whose percentage contribution (in parenthesis) to the corresponding Orbach transition rate is above the given threshold are displayed.

Fortran codes to calculate CFPs thermal evolution

In the folder Home-Made-Codes one finds the codes cfptemp.f and cfppert.f. The input is frrmcfpd2.inp in both cases (see the section "Fortran code to evaluate magnetic relaxation dynamics").

cfptemp.f: this code calculates the CFPs $A_k^q\left\langle r^k\right\rangle(T)$ in cm^{-1} at a given temperature T (see the file CFPs-vs-Temp). To set the temperature, open the code and change the variable temp (K), where the number of vibrational modes is also found. The output is cfptemp.out. In this output, the CFPs at the relaxed geometry, at T = 0 K and at the given temperature are printed. The library lapack is not required. To compile, we use the compiler gfortran and the command line: gfortran –o aa cfptemp.f (aa is the executable name).

cfppert.f: this code calculates the parameters $\Delta\left(A_k^q\left\langle r^k\right\rangle\right)_j(T)$ in cm^{-1} (see Eq. 1 in main text) for all vibrational modes j at a given temperature T (see the folder cfppert-temp). To set the temperature, open the code and change the variable temp (K), where the number of vibrational modes is also found. The output is cfppert.out. In this output, the parameters $\Delta\left(A_k^q\left\langle r^k\right\rangle\right)_j(T)$ are printed for each vibrational mode. These parameters are printed in two columns. In the left column and from top to bottom, they appear as: (2,0), (2,1), (2,2), (4,0), (4,1), (4,2), (4,3), (4,4), (6,0), (6,1), (6,2), (6,3), (6,4), (6,5), (6,6). In the right column and from top to bottom they appear as: nothing(always 0.00000000), (2,-1), (2,-2), nothing(always 0.00000000), (4,-1), (4,-2), (4,-3), (4,-4), nothing(always 0.00000000), (6,-1), (6,-2), (6,-3), (6,-4),

(6,-5), (6,-6). In these parenthesis, the first number is the script k = 2, 4, 6, while the second number is the script q = -k,…, +k. Now, we use cfppert.out as an input for the code cfptomat.f (which can also be found in the folder Home-Made-Codes) in order to generate the perturbing Hamiltonians $\hat{H}_j$ that appear in the input matrix.inp (see section "Fortran code to evaluate magnetic relaxation dynamics"). Inside the code cfptomat.f one finds the following variables: inn, which selects the magnetic metal ion; dj, which is the metal ground electron spin quantum number J, idtot, which is $2J+1$ and has also to be changed when needed in the function oplm and in the subroutine operators; nmod, which is the number of vibrational modes. The library lapack is not required. To compile, we use the compiler gfortran and the command lines: gfortran –o aa cfppert.f and gfortran –o aa cfptomat.f (aa is the executable name).

Description of the REC (Radial Effective Charge) model, and generation of an initial guess for the effective charges and the effective radial distances

The REC model is an electrostatic *semi-empirical* model commonly used in molecular magnetism, which provides an estimation of the crystal field parameters (CFPs) and allows rationalizing the magnetic properties of a given *f*-block single-ion magnetic coordination compound.(ref. 18 in main text) From the calculated CFPs, the model gives the ground-*J* multiplet energy levels and their corresponding wave-functions as a linear combination of the different m_J = -J,…, +J microstates. For that, the (crystallographic) atomic coordinates of the first coordination sphere are required as an input. The REC model is implemented in the portable *fortran* SIMPRE computational package.([4] and ref. 11 in main text) This code parameterizes the electric field effect produced by the coordinating ligands by using the Crystal Field Hamiltonian in Eq. S8, expressed in terms of the Stevens Equivalent Operators (SEOs)[5]:

$$\hat{H}(J) = \sum_{k=2,4,6}\sum_{q=-k}^{+k} B_k^q \hat{O}_k^q = \sum_{k=2,4,6}\sum_{q=-k}^{k} (1-\sigma_k) A_k^q \langle r^k \rangle \eta_k \hat{O}_k^q \quad \text{Eq. S8}$$

In Eq. S8, *k* is the order (also called rank or degree) and *q* is the range, which varies between *k* and *–k*, of the SEOs $\hat{O}_k^q$. These are defined by Ryabov in terms of the angular momentum operators $J_\pm$ and J_z,[6] where the components $\hat{O}_k^q(c)$ and $\hat{O}_k^q(s)$ correspond to the SEOs with q ≥ 0 and q < 0 respectively.[7] Note that all the Stevens CFPs $A_k^q \langle r^k \rangle$ are real, whereas the matrix elements of $\hat{O}_k^q$ (q < O) are imaginary. η_k are the α, β and γ Stevens coefficients for *k* = 2, 4, 6, respectively, which are tabulated and depend on the number of *f* electrons.[8] σ_k are the Sternheimer shielding parameters of the 4*f* electronic shell,[7] and $\langle r^k \rangle$ are the expectation values of the radius k^{th} power.[9]

In SIMPRE, the A_k^q parameters are determined by the following relations (second relation when q>0, third relation when q<0):

$$A_k^0 = \frac{4\pi}{2k+1}\sum_{i=1}^{N}\frac{Z_i e^2}{R_i^{k+1}} Z_{k0}(\theta_i,\varphi_i) p_{kq}$$

$$A_k^q = \frac{4\pi}{2k+1}\sum_{i=1}^{N}\frac{Z_i e^2}{R_i^{k+1}} Z_{kq}^c(\theta_i,\varphi_i) p_{kq}$$

$$A_k^q = \frac{4\pi}{2k+1}\sum_{i=1}^{N}\frac{Z_i e^2}{R_i^{k+1}} Z_{k|q|}^s(\theta_i,\varphi_i) p_{k|q|}$$

Eq. S9

(q>0)

In Eq. S9, R_i, θ_i and φ_i are the effective polar coordinates of the point charges, and Z_i is the magnitude of the effective point charge, associated to the *i*-th donor atom with the lanthanide ion at the coordinate origin, *N* is the number of ligands; *e* is the electron charge, p_{kq} are the prefactors of the spherical harmonics and Z_{kq} are the tesseral harmonics expressed in terms of the polar coordinates for the i-th donor atom.

In the REC model, any given ligand is modeled as an effective point charge placed between the lanthanide ion and the ligand coordinating atom at a distance R_{eff} from the lanthanide ion, which is smaller than the real lanthanide-ion-donor-atom distance (r_i).(ref. 18 in main text) To account for the effect of covalency, a radial displacement vector (D_r) is defined, in which the polar coordinate *r* of each coordinating atom is collectively varied as $R_{eff} = r_i - D_r$, and at the same time the charge magnitude (Z_i) is scanned in order to achieve a minimum deviation between the calculated and the experimental target property P (e.g. the ground-*J* multiplet energy levels), whereas θ_i and φ_i remain constant (see Fig. S1).

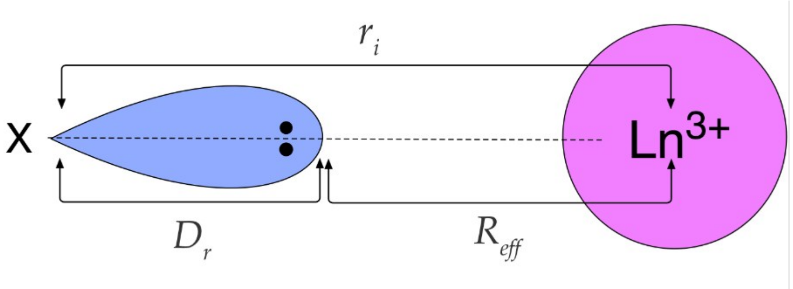

Figure S1: Lone electronic pair of a donor atom X oriented towards the nucleus of a trivalent lanthanide ion. The effective charge is located between the lanthanide ion and X at $R_{eff} = r_i - D_r$.

As a starting point for the fitting, we can estimate the effective distances of the coordinating atoms by using the following *semi-empirical* approximation for D_r :(ref. 19 in main text)

$$D_r \approx \left(\frac{N_L}{V_M}\right)\frac{1}{E_M\left(E_L - E_M\right)} \quad \text{Eq. S10}$$

In Eq. S10, N_L is the coordination number of the lanthanide ion, V_M is the valence of the lanthanide ion, and E_M and E_L are the Pauling electronegativity of the lanthanide ion and the donor atom, respectively. The effective charge Z_i is estimated by using Eq. S11:

$$f_{X,CN} = D_r Z_i \quad \text{Eq. S11}$$

In Eq. S11, *f* is a factor that depends on the coordination number (CN) and on the coordinating atom (X).(ref. 19 in main text) The use of Eq. S11 is limited, since its use for a given system requires to know *f* in advance and, for that, one first has to know the REC parameters of a large

enough set of coordination compounds with different lanthanide ion but with the same or similar ligands with the same coordination number.

In order to obtain the REC parameters (D_r and Z_i) of the target compound, we need to vary both of them until a satisfactory fitting of a given property P –either experimental (e.g. spectroscopic energy levels, spectroscopically-determined CFPs, or magnetic properties) or calculated (e.g. via *ab initio* calculations)– is achieved. In the case of lanthanide single-ion coordination compounds, a fit of the ground-*J* multiplet energy levels will always be the desired option. Unfortunately, we cannot extrapolate this procedure to actinides, where the effects of excited multiplets are more important and thus are not negligible at all. In that case, in SIMPRE, one can fit the CASSCF or spectroscopically-determined CFPs using the REC model, and then obtain the energy level scheme by using the full Hamiltonian in the CONDON package.(ref. 20 in main text) The so-called *full model* therein considers inter-electronic repulsion, spin-orbit coupling, the ligand field potential, and, of course, both ground and excited J multiplets.[10] In the fitting procedures, we define the relative error *E* as:

$$E = \frac{1}{n}\sum_{i=1}^{n}\frac{\left[P_{fit,i} - P_{ref,i}\right]^2}{\left[P_{ref,i}\right]^2} \quad \text{Eq. S12}$$

In Eq. S12, P_{ref} is the relevant property to fit and P_{fit} is the best fit to P_{ref}, and *n* is the number of points used in the fiting.

Projection on $[U(Cp^{ttt})_2]^+$ of the CASSCF energies evaluated at the $[Dy(Cp^{ttt})_2]^+$ experimental geometry

To determine the REC parameters that describe the crystal field produced by the two coordinating Cp^{ttt} = $\{C_5H_2{}^tBu_3\text{-}1,2,4\}$ rings in $[U(Cp^{ttt})_2]^+$, our starting point will be the CASSCF energy levels determined by Goodwin *et al.* at the experimental geometry of $[Dy(Cp^{ttt})_2]^+$.(ref. 3 in main text) The experimental crystallographic coordinates of the first coordination sphere of $[Dy(Cp^{ttt})_2]^+$ are used as an input in SIMPRE, and the two REC parameters are varied to fit the calculated CASSCF energy levels at the experimental geometry of $[Dy(Cp^{ttt})_2]^+$. By using the REC model, the best fit, with an error of E = 0.03 % (Eq. S12), results in D_r = 1.313 Å and Z_i = 0.068. The calculated ground *J* multiplet energy levels (E_{fit}) by SIMPRE with these REC parameters are compared with the CASSCF ones (E_{ref}) in Table S2.

Table S2: Ground-*J* multiplet Kramers doublets determined by CASSCF (E_{ref}) and by the REC model (E_{fit}) for $[Dy(Cp^{ttt})_2]^+$. $\Delta E = |E_{ref} - E_{fit}|$. Relative errors are < 2.6 %.

E_{ref}–CASSCF (cm^{-1})	E_{fit}–REC (cm^{-1})	ΔE (cm^{-1})

0	0	–
488.6	480.7	7.9
771.0	775.6	4.6
956.5	980.9	24.4
1122.2	1145.4	23.2
1277.5	1280.5	3.0
1399.3	1365.4	33.9
1476.1	1465.4	10.7

Subsequently, we apply these calculated REC parameters to the DFT-relaxed coordinates of the coordinating atoms in $[U(Cp^{ttt})_2]^+$. This target compound has identical ligands as $[Dy(Cp^{ttt})_2]^+$ and only the metal ion is different. This allows us to transfer the REC parameters from $[Dy(Cp^{ttt})_2]^+$ to $[U(Cp^{ttt})_2]^+$ as demonstrated in several works.([11,12] and refs. 17, 19 in main text) The input coordinates of the relaxed positions of the coordinating atoms in $[U(Cp^{ttt})_2]^+$ (*simpre.dat* file) and the calculated CFPs (*simpre.out*) are reported in Table S3 and Table S4, respectively. This procedure is systematically repeated for each distorted geometry along each vibrational mode, and the corresponding set of CFPs is obtained by performing a millisecond calculation in SIMPRE.

Table S3: Relaxed input coordinates of the coordinating atoms in $[U(Cp^{ttt})_2]^+$ after applying D_r = 1.313 Å to the radial coordinate and using a magnitude charge of Z_i = 0.068.

Label	R_{eff} (Å)	θ (°)	ϕ (°)	Z_i
C1	1.3017302	16.9734964	170.7902831	0.06806
C2	1.3061774	15.4279100	349.0185755	0.06806
C3	1.3907467	39.0491022	39.7965899	0.06806
C4	1.4903647	49.2710181	80.8124802	0.06806
C5	1.3744865	40.4952024	122.0849379	0.06806
C6	1.3016208	163.0437971	63.0361018	0.06806
C7	1.3061446	164.5540736	244.7622728	0.06806
C8	1.3908882	140.9353178	194.0198766	0.06806
C9	1.4905911	130.7250294	153.0093834	0.06806
C10	1.3745142	139.5118956	111.7435627	0.06806

Table S4: Calculated CFPs for the $[U(Cp^{ttt})_2]^+$ DFT-relaxed geometry in Stevens ($A_k^q \left\langle r^k \right\rangle$ and B_k^q) and Wybourne (B_{kq}) notation.

k	***q***	$A_k^q\langle r^k\rangle$ (cm^{-1})	B_k^q (cm^{-1})	B_{kq} (cm^{-1})
2	0	997.8	-6.414	1995.6
2	1	-1118.3	7.188	-456.5
2	-1	-568.0	3.651	-231.9
2	2	-47.1	0.303	-38.5
2	-2	-64.4	0.414	-52.6
4	0	262.0	-0.076	2095.8
4	1	-623.1	0.181	-557.3
4	-1	-316.2	0.092	-282.8
4	2	-52.3	0.015	-66.1
4	-2	-71.1	0.021	-89.9
4	3	-36.9	0.011	-12.5
4	-3	-228.9	0.067	-77.4
4	4	-30.2	0.009	-28.9
4	-4	95.2	-0.028	91.0
6	0	67.4	-0.003	1079.1
6	1	823.0	-0.031	1016.0
6	-1	419.4	-0.016	517.7
6	2	374.3	-0.014	584.5
6	-2	512.9	-0.019	800.9
6	3	-202.4	0.008	-158.0
6	-3	-1254.1	0.048	-979.1
6	4	-184.9	0.007	-117.9
6	-4	581.4	-0.022	370.6
6	5	-98.3	0.004	-29.9
6	-5	98.6	-0.004	30.0
6	6	-50.6	0.002	-53.3
6	-6	17.1	-0.001	18.0

Finally, the CFPs in Wybourne notation of the $[U(Cp^{ttt})_2]^+$ DFT-relaxed geometry are used as an input in the CONDON package to determine the equilibrium electronic structure of $[U(Cp^{ttt})_2]^+$,

reported in Fig. 1. The wave-functions as determined by CONDON are expressed as a linear combination of the several J multiplets (both ground and excited). We truncate them to the ground J multiplet and then renormalize the resulting expression.

Determination of the REC parameters for the uranium-based SIM UTp_3

To determine the two REC parameters in UTp_3 we start from its lowest 10 experimental energies -in the form of 5 degenerate Kramers doublets- reported in ref. 13. As explained in the step 2 of our methodology described in the main text, we first run a SIMPRE calculation on the experimental $NdTp_3$ geometry with Z_i = 0.06806 to determine an initial guess of CFPs. These CFPs are now introduced in CONDON with Wybourne notation to extract the corresponding lowest 10 energies but by selecting U^{3+} in the input instead of Nd^{3+}. This process is iteratively repeated until obtaining a satisfactory match between the calculated energies by CONDON and the experimental ones. The REC parameters determined are D_r = 1.414 Å and Z_i = 0.0305. The ground *J* multiplet energy levels (E_{fit}) calculated by CONDON with these parameters are compared with the experimental ones (E_{ref}) in Table S5.

Table S5: Ground-*J* multiplet Kramers doublets determined experimentally (E_{ref}) and by the REC+CONDON method (E_{fit}) for UTp_3. ΔE = |E_{ref} - E_{fit}|. Relative errors are < 11.0 %.

E_{ref}-exp (cm^{-1})	E_{fit}-REC+CONDON(cm^{-1})	ΔE (cm^{-1})
0	0	-
270	268	2
567	574	7
693	617	76
805	717	88

Table S6: Relaxed input coordinates of the coordinating atoms in UTp_3 after applying D_r = 1.414 Å to the radial coordinate and using a magnitude charge of Z_i = 0.0305.

Label	R_{eff} (Å)	θ (°)	ϕ (°)	Z_i
N1	1.3391597	90.1072662	329.9395403	0.0305
N2	1.3385925	90.0922733	89.9405456	0.0305
N3	1.3387928	90.0696210	209.9420622	0.0305
N4	1.1604032	51.1959304	30.7005972	0.0305
N5	1.1601574	51.1974414	150.6905931	0.0305
N6	1.1603399	51.1800165	270.7172309	0.0305
N7	1.1558034	128.5308149	31.2350147	0.0305
N8	1.1554881	128.5508786	151.2494470	0.0305
N9	1.1558833	128.5303373	271.2252646	0.0305

Table S7: Calculated CFPs for the UTp_3 DFT-relaxed geometry in Stevens ($A_k^q \langle r^k \rangle$ and B_k^q) and Wybourne (B_{kq}) notation.

k	***q***	$A_k^q \langle r^k \rangle$ (cm^{-1})	B_k^q (cm^{-1})	B_{kq} (cm^{-1})
2	0	-42.57383674	0.27366103	-85.14767347
2	1	-0.06629834	0.00042616	-0.02706619
2	-1	0.10233866	-0.00065782	0.04177958
2	2	0.00924865	-0.00005945	0.00755149
2	-2	-0.01272683	0.00008181	-0.01039141
4	0	-264.11623947	0.07688628	-2112.92991577
4	1	-1.18194763	0.00034407	-1.05716610
4	-1	0.90891315	-0.00026459	0.81295664
4	2	0.69564707	-0.00020251	0.87993167
4	-2	-1.19923537	0.00034911	-1.51692609
4	3	-121.08610621	0.03524910	-40.93457514
4	-3	87.95434454	-0.02560419	29.73399540
4	4	0.09552924	-0.00002781	0.09134343
4	-4	0.12123624	-0.00003529	0.11592402
6	0	36.89099621	-0.00140141	590.25593936
6	1	1.72507498	-0.00006553	2.12947878
6	-1	-0.97013783	0.00003685	-1.19756414
6	2	-1.00210675	0.00003807	-1.56472968
6	-2	0.33870143	-0.00001287	0.52886200
6	3	-50.15855746	0.00190542	-39.15979191
6	-3	-5.73091273	0.00021771	-4.47423852
6	4	-1.06653956	0.00004052	-0.67987125
6	-4	-1.88420231	0.00007158	-1.20109467
6	5	-9.70898835	0.00036882	-2.95051176
6	-5	2.89640123	-0.00011003	0.88020148
6	6	-2110.50736207	0.08017387	-2221.77903375
6	-6	-114.21442851	0.00433877	-120.23612292

References

[1] Gaussian09, Revision D.01, M. J. Frisch, G. W. Trucks, H. B. Schlegel, G. E. Scuseria, M. A. Robb, J. R. Cheeseman, G. Scalmani, V. Barone, B. Mennucci, G. A. Petersson, H. Nakatsuji, M. Caricato, X. Li, H. P. Hratchian, A. F. Izmaylov, J. Bloino, G. Zheng, J. L. Sonnenberg, M. Hada, M. Ehara, K. Toyota, R. Fukuda, J. Hasegawa, M. Ishida, T. Nakajima, Y. Honda, O. Kitao, H. Nakai, T. Vreven, J. A. Montgomery, Jr., J. E. Peralta, F. Ogliaro, M. Bearpark, J. J. Heyd, E. Brothers, K. N. Kudin, V. N. Staroverov, T. Keith, R. Kobayashi, J. Normand, K. Raghavachari, A. Rendell, J. C. Burant, S. S. Iyengar, J. Tomasi, M. Cossi, N. Rega, J. M. Millam, M. Klene, J. E. Knox, J. B. Cross, V. Bakken, C. Adamo, J. Jaramillo, R. Gomperts, R. E. Stratmann, O. Yazyev, A. J. Austin, R. Cammi, C. Pomelli, J. W. Ochterski, R. L. Martin, K. Morokuma, V. G. Zakrzewski, G. A. Voth, P. Salvador, J. J. Dannenberg, S. Dapprich, A. D. Daniels, O. Farkas, J. B. Foresman, J. V. Ortiz, J. Cioslowski, and D. J. Fox, Gaussian, Inc., Wallingford CT, 2013.

[2] Introduction to lattice dynamics. Dove, M. T. Cambridge University Press (1993).

[3] Molecular magnetism. Kahn, O. VCH Publishers, Inc. (1993).

[4] J. J. Baldoví, S. Cardona-Serra, J. M. Clemente-Juan, E. Coronado, A. Gaita-Ariño, A. Palii, *J. Comput. Chem.,* **2013**, 34, 1961.

[5] (a) C. Rudowicz, C. Y. Chung, *J. Phys. Condens. Matter*, **2004**, 16, 5825; (b) C. Rudowicz, *J. Phys. C: Solid State Phys.*, **1985**, 18, 1415; (c) C. Rudowicz, *J. Phys. C: Solid State Phys*., **1985**, 18, 3837 (erratum).

[6] I. D. Ryabov, *Journal of Magnetic Resonance*, **1999***,* 140, 141.

[7] S. Edvardsson, M. Klinterberg, *Journal of Alloys and Compounds,* **1998**, 275, 233.

[8] K. W. H. Stevens, *Proc.Phys. Soc.* **1952**, *65*, 209.

[9] M. Speldrich, J. van Leusen, P. Kögerler, *J. Comput. Chem.* **2018**, 39, 2133.

[10] M. Speldrich, J. van Leusen, P. Kögerler, *J. Comput. Chem.* **2018**, 39, 2133.

[11] J. J. Baldoví, Juan M. Clemente-Juan, A. Gaita-Ariño, E. Coronado*, Inorg. Chem.* **2014**, 53(20), 11323.

[12] J. J. Baldoví, Y. Duan, R. Morales, A. Gaita-Ariño, E. Ruiz, E. Coronado, *Chem.–Eur. J.*, **2016**, 22, 13532.

[13] Amberger et al., *Z. Anorg. Allg. Chem.*, **2010**, 636, 201.
