# Supplementary material for: Design of high-temperature f-block molecular nanomagnets through the control of vibration-induced spin relaxation
Source: Chem Sci. 2019 Dec 2;11(6):1593–8. doi: 10.1039/c9sc03133b (PMC7025469; doi:10.1039/c9sc03133b)
Supplement: Supplementary file 2 [file SC-011-C9SC03133B-s002.pdf]

Electronic Supplementary Material (ESI) for Chemical Science.
This journal is © The Royal Society of Chemistry 2020

Efficient design of High-temperature Single-molecule Magnets

Single-molecule magnets (SMMs) represent the limit of miniaturization and are attractive potential candidates for information storage

A major roadblock for their practical application is their cryogenic working temperature

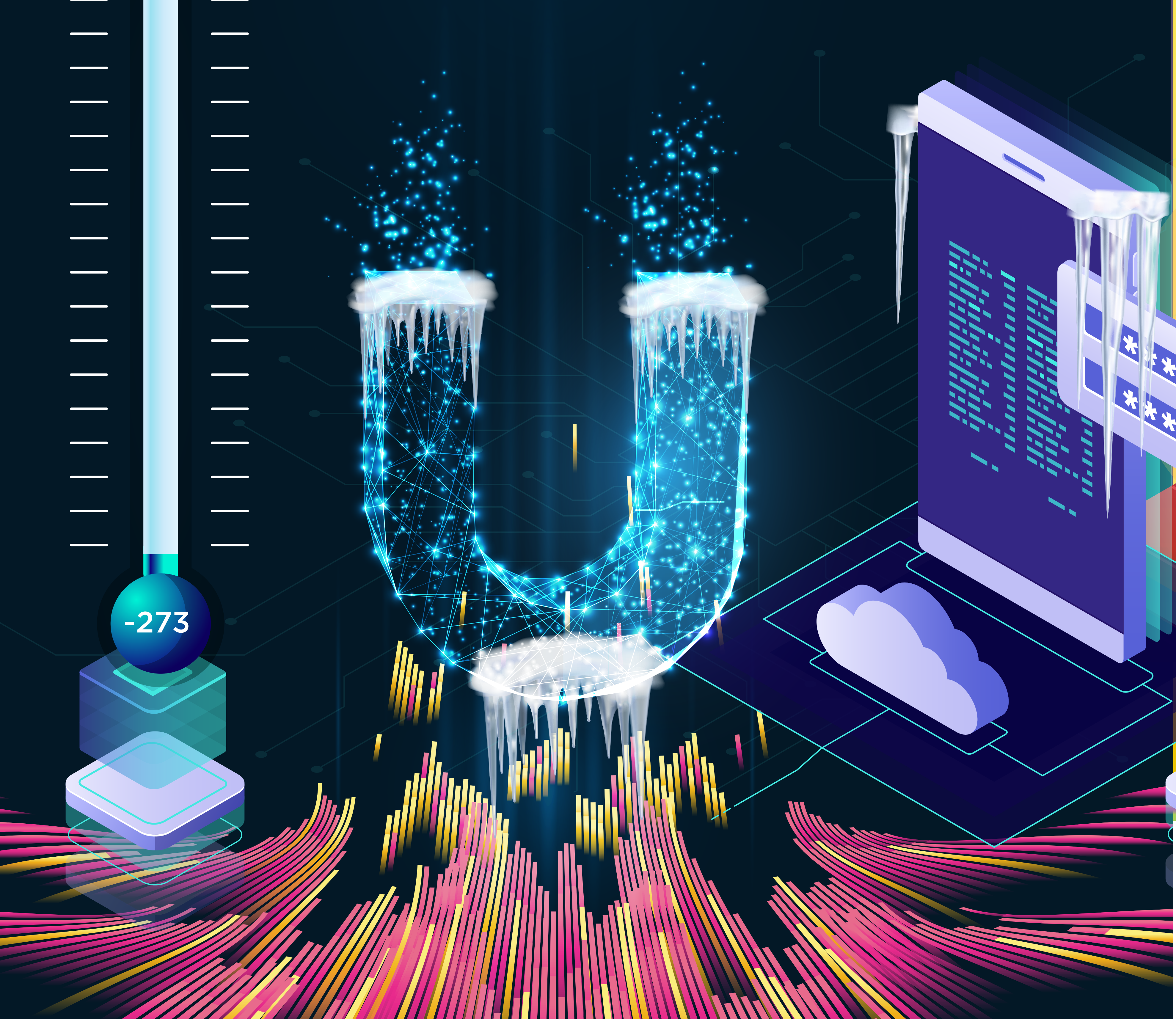

An efficient methodology to explore and chemically design new SMMs with higher operating temperatures

First-principles approach to simulate spin relaxation in f-block SMMs

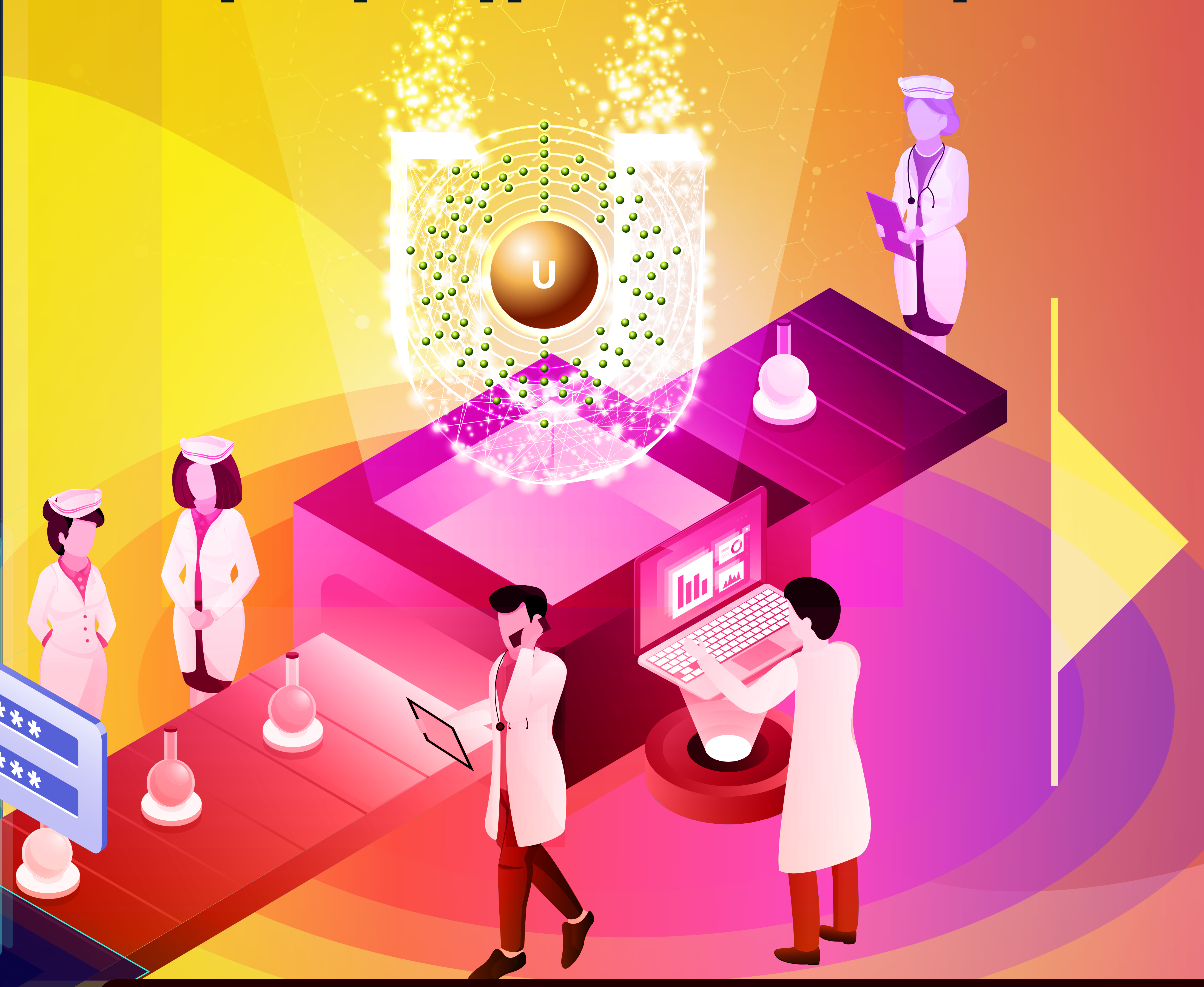

- Just one Complete Active Space Self Consistent Field calculation
- Faster than state-of-the-art methods
- Accurate and practical
- Thermal dependence of relaxation pathways

Theoretical tools reported could be used to design and predict the behaviour of SMMs that can function at higher temperatures

Chemical Science

PICK OF THE WEEK

Design of high-temperature f-block molecular nanomagnets through the control of vibration-induced spin relaxation

Baldoví *et al.* (2019) | DOI: 10.1039/C9SC03133B

ROYAL SOCIETY OF CHEMISTRY
